# Supplementary material for: The temporality of uncertainty in decision-making and treatment of severe brain injury
Source: PLoS One. 2020 Oct 1;15(10):e0238506. doi: 10.1371/journal.pone.0238506 (PMC7529300; doi:10.1371/journal.pone.0238506)
Supplement: S1 File — (DOCX) [file pone.0238506.s001.docx]

Internals\\Empirisk data\\1. marts. Logopæder og målsætningsmøde - § 2 references coded [5.51% Coverage]

Reference 1 - 3.26% Coverage

Der står foruden på tavlen, at det aktuelt er 21 timer og 25 min. siden patientens ankomst til NISA. Alle patienterne har en note om, hvor længe de har været indlagt på NISA. På tavlen ser jeg også, at der ud for flere patienter står "logopæd", hvilket også er tilfældet ud for patienten N, som jeg følger.

*It also states at the board that it is currently 21 hours and 25 minutes since the patient's arrival at NISU. All patients have a note beside their name stating how long they have been hospitalized at NISU. On the board that next to several patients is the word "speech therapist", which is also the case with patient N, whom I observe.*

Reference 2 - 2.26% Coverage

Da alle er samlet til morgen briefing gennemgår sygeplejerskerne deres patienter for lægerne, hinanden og terapeuterne. Patient K. ligger på stue 2, hvorfor hun bliver nummer to som gives en briefing på.

*As everyone is gathered for the morning briefing, the nurses review their patients for the doctors, each other, and the therapists. Patient K. is in room 2, for which reason the briefing of her status is the second in line.*

Internals\\Empirisk data\\10. feb. Observation af første træning for N, 1. forløb - § 4 references coded [ 5.14% Coverage]

Reference 1 - 1.50% Coverage

Sygeplejersken er ansvarlig i dag for, at det hele ”spiller” – hvilket er en del af specialuddannelsen, som intensiv sygeplejerske skal lære i denne del af arbejdet også.

*The nurse is responsible for everything “play” today - which is part of the special training as an intensive care nurse that she must learn in this part of the work as well.*

Reference 2 - 1.50% Coverage

Hvor det i dag er terapeuter fra Hammel Neurocenter, som står for genoptræningen på NISA, var det tidligere interne terapeuter fra Silkeborg hospital, som stod for genoptræningen.

*Where today it is therapists from Hammel Neurocentre who are responsible for the rehabilitation at NISU, it was former internal therapists from Silkeborg hospital who were responsible for the rehabilitation.*

Reference 3 - 0.67% Coverage

Den intensive afdeling består af: 6 NISA senge + 2 medicinske intensive senge + 1 akut seng

*The intensive care unit consists of: 6 NISU beds + 2 medical intensive care beds + 1 emergency bed*

Reference 4 - 1.47% Coverage

”Det spændende ved denne afdeling er det skiftende”. Det langsomme og stille (typisk NISA) – så det hurtige, hvor der sker en hel masse lige pludseligt (intensive patienter).

*“The exciting thing about this department is the changing": The slow and quiet (typically NISU) – and then the fast and a whole lot happen all of a sudden (intensive patients).*

Internals\\Empirisk data\\13. feb. Fys træning m. patient N - § 1 reference coded [ 4.54% Coverage]

Reference 1 - 4.54% Coverage

Sygeplejersken siger, at nogle steder får patienter ikke træning, før de har åbnet øjnene og har øjnerespons (hvilket slet ikke er godt). Der er dog ofte nogle strækøvelser (kan ikke huske, hvad de kaldte dem). ”Det der gør os særlige her, er at vi giver genoptræning fra starten af”.

*The nurse says that in some places, patients do not get exercise until they have opened their eyes and have eye response (which is not good at all). However, there are often some stretching exercises (I can't remember what they called them). "What makes us special here is that we provide rehabilitation from the start”.*

Internals\\Empirisk data\\14. feb. modtagelse af ny patient (P. – 2. forløb) - § 4 references coded [ 5.80% Coverage]

Reference 1 - 2.58% Coverage

Sygeplejerske: Jo før de medicinske patienter kommer her jo bedre, da de ikke får samme tidlige neurorehabilitering andre steder. Ellers kan de ligge på intensivafdelinger i flere måneder på hospitaler. Det er ikke godt.

*Nurse: The sooner the medical patients come here the better, as they do not get the same early neuro-rehabilitation elsewhere. Otherwise, they can stay in intensive care units for several months in hospitals. It is not good.*

Reference 2 - 0.27% Coverage

Middagskonferencen kl. 13

*Conference at 1 pm.*

Reference 3 - 0.11% Coverage

Rehab.plan

*Rehabilitation plan*

Reference 4 - 2.84% Coverage

NISA er modsat de hospitaler, hvor patienten ligges til at sove ved medicin, da NISA netop arbejder på at vække patienterne op. På NISA er metoden at finde og afprøve forskellige stimuli, der gør at patienten vågner (fx duft, lyde, bevægelse).

*NISU is the opposite of the kind of hospitals where the patient is put to sleep by medication, as NISA is working to wake up the patients. At NISU, the method is to find and test different stimuli that make the patient wake up (that is smell, sounds, movement).*

Internals\\Empirisk data\\16 feb. Målsætningssamtale pårørende til N. + Indscore - § 2 references coded [ 2.03% Coverage]

Reference 1 - 0.09% Coverage

”Suction Aid”

*”Suction Aid”*

Reference 2 - 1.93% Coverage

Muligheden for at kunne suge ud er meget vigtigt, forklarer sygeplejersken, i forhold til genoptræning. Der må nemlig ikke ligge en ”sø” (af slim, snot, mundvand) oven over ”cuffen” (den lille luftballon som bliver pustet op nede i halsen og således lukker for, at spyt etc. kan løbe ned i luftvejene/lungerne.

*The option of being able to “suck out” is very important in relation to rehabilitation, the nurse explains. There must not be a "lake" (of mucus, mouthwash) above “the cuff" (the small balloon which is inflated down the throat and thus closes so that saliva etc. can not run down into the airways/lungs.*

Internals\\Empirisk data\\20 feb. Praktikant og pårørendemøde - § 4 references coded [ 3.43% Coverage]

Reference 1 - 2.51% Coverage

Praktikanten (sygeplejersken) undrer sig over måden, de har vagtskifte på her i forhold til hospitalet H, hvor de overlapper hinanden og mundtligt fortæller og giver status. Udviklingssygeplejersken fortæller: ”Der er ingen mundtlig overlevering her”. Hun fortæller, at dette gør de sådan, fordi der er forskel på det, der bliver sagt og hvad der høres – og hvis en information er vigtig, skal den skrives ned.

*The trainee (nurse) wonders about the way they at NISU have change in shifts here compared to Hospital H, where they overlap and verbally tell and give status. The developmental nurse says: "There is no oral delivery here". She says that they do this because there is a difference between what is said and what is heard - and if information is important, it must be written down.*

Reference 2 - 0.20% Coverage

”tværfaglig planlægning”

*"Interdisciplinary planning"*

Reference 3 - 0.31% Coverage

Her at NISA har terapeuterne en mening til tuben.

*Here at NISU, the therapists have an opinion for the tube.*

Reference 4 - 0.41% Coverage

Kl. 8.10 samler alle sig omkring skænken med computere.

*At 8.10 everyone gathers around the sideboard where the computers are placed.*

Internals\\Empirisk data\\21. feb. Røntgen og FEES undersøgelse - § 2 references coded [ 1.73% Coverage]

Reference 1 - 0.20% Coverage

FEES-undersøgelsen

*FEES- examination*

Internals\\Empirisk data\\24. jan. Fysioterapeut - § 6 references coded [ 7.04% Coverage]

Reference 1 - 0.70% Coverage

Det er ikke til at vide sig sikker på dagen inden, hvordan det ser ud om morgenen.

*It is never to know for sure the day before what it looks like in the morning.*

Reference 2 - 0.51% Coverage

Dernæst går vi hen til endnu (den tredje) NISA-patient,

*Next, we go to yet another (third) NISU-patient,*

Reference 3 - 2.36% Coverage

Hun kan gå, som den eneste af de patienter jeg har observeret indtil nu. Hun har været på afsnittet en gang før, kom så til Hammel, men er atter tilbage på NISA i Silkeborg, fordi hendes hals har dannet meget arvæv indeni efter tuben blev fjernet. Hun trækker derfor med besvær vejret.

*She can walk, as the only one of the patients I have observed so far. She has been to the unit once before, then she was moved to Hammel, but is yet back at NISU in Silkeborg, because her inside her throat has formed a lot of scar tissue after the tube was removed. She therefore breathes with difficulty.*

Reference 4 - 0.88% Coverage

”Tavlen”: Det er en ny ting, at tavlen med de røde streger er hængt op for at give overblik over dagens program

*"The board": It is a new thing that the board with the red lines is hung up to give an overview of today's program*

Reference 5 - 1.36% Coverage

Tavlen: Det er meningen, at lægen skal bruge de små røde eller grønne magneter for at vise, hvor han har/har været på stuegang, og at de andre faggrupper skriver på, hvad de gør, fx fys-træning.

*The board: It is intended that the doctor shall use the small red or green magnets to show where he is/has been on ward rounds, and that the other professional groups write what they do next to each patient (e.g. physical training).*

Reference 6 - 1.23% Coverage

Afdelingssygeplejersken og jeg aftaler, at jeg møder tidligt ind dagen efter og ser vagtskiftet samt morgenmødet. Hun siger, der er god dynamik heri.

*The ward nurse and I agree that I will show up early the next day and see the shift change and the morning meeting. She says there is good dynamism in this.*

Internals\\Empirisk data\\25. jan. En dag med erfaren sygepl. - § 3 references coded [ 4.29% Coverage]

Reference 1 - 0.51% Coverage

morgenmødet som sker klokken 8.15 hver morgen.

*the morning meeting which takes place at 8.15 every morning.*

Reference 2 - 2.80% Coverage

Sygeplejersken fortæller, at det er helt uvant for hende at skulle servere mad for sine patienter, men patienten hun er på i dag, er meget atypisk for NISA. Hun venter egentligt på at komme videre ud til Hammel, men der er ikke plads.

*The nurse tells me that it is quite unusual for her to have to serve food to her patients. However, the patient she is on today is very atypical of NISU - she is in fact just waiting to be moved on to Hammel, but there is no room.*

Reference 3 - 0.98% Coverage

hver dag er det den ansvarshavende sygeplejerske, der har vagten, som deler patienterne ud imellem sygeplejerskerne.

*every day, it is the nurse in charge, who has the guard, who distributes the patients among the nurses.*

Internals\\Empirisk data\\27 feb - Udskrivelse af P - § 3 references coded [ 3.43% Coverage]

Reference 1 - 0.24% Coverage

FOTT betegner synketræning

*FOTT has to do with swallowing training*

Reference 2 - 2.94% Coverage

Patienter, som kommer fra Regionmidt hører alle til NISA. Patienter ude fra andre regioner: Her skal deres region selv betale for at have en patient til Hammel eller NISA. Det giver derfor økonomisk noget for Silkeborg Sygehus at få en patient fra fx RegionNord, hvilket ikke er tilfældet med patienter fra RegionMidt, da de allerede er en del af deres budget

*Patients who come from Regionmidt all belong to NISU. Patients from other regions: Here the region must pay to have a patient send to Hammel or NISU. It therefore gives something financial for Silkeborg Hospital to get a patient from e.g. RegionNord, which is not the case with patients from RegionMidt, as they are already part of their budget*

Reference 3 - 0.25% Coverage

neuro-pædagogiske strategi

*neuro pedagogical strategy*

Internals\\Empirisk data\\27 marts (aftenvagt) - § 9 references coded [ 10.36% Coverage]

Reference 1 - 2.76% Coverage

Jeg tænker, at jeg gerne vil se, hvordan vagtskiftet foregår, hvorfor jeg har skyndt mig at klæde om og komme ind på afdelingen. Der er altid et tidligt vagtskifte og et sent vagtskifte hold, hvor det tidlige kommer en halv time før – om aftenen møder de ind klokken 15, og det sene hold møder ind klokken 15.30. Jeg går en runde på N1 for at danne mig et overblik. Det er lidt svært at gennemskue for mig, hvem som er på vej ind og hvem som er på vej hjem.

*I would like to see how the shift changes, why I have hurried to change to the uniform and get into the unit. There is always an early shift change and a late shift change, where the early ones arrive half an hour before - in the evening they arrive at 3 pm, and the late shift arrives at 3.30 pm. I walk around on the N1 to get an overview. It's a little hard for me to figure out who's going in and who's going home.*

Reference 2 - 1.20% Coverage

Uden foran sidder en ung sygeplejerske (lyshåret). Hun fortæller, at hun har patienten på stue 8 i aften, som hun er i gang med at læse op på i EPJ, og skriver noter ned på et stykke papir imens.

*Outside sits a young nurse (blond). She says that she has the patient in room 8 tonight, whom she is reading about in* EPJ *(Electric patient Journal). She is writing down notes on a piece of paper.*

Reference 3 - 2.40% Coverage

The nurse mødte også ind klokken 15. Hun har EPJ åben og læser op på sine to patienter. Hun tjekker, ”at der ikke er noget på patienterne, der bimler og bamler”. Hun forklarer mig, at hun nederst i notatet på EPJ kan læse, om der står skrevet noget som hun skal være opmærksom på at klare med det samme, fx hvis en patient ikke havde fået sin medicin kl. 14 – men det er der ikke i dag.

*The nurse arrives to the unit at 3pm as well. She has EPJ open and is reading about the two patients given to her. She checks, "that there is nothing on the patients who “bumble and bumble". She explains to me that at the bottom of EPJ is a note where she can read if something has been written about the patient that she must be aware of and deal with immediately, e.g. if a patient had not received his medication at 2 pm. But this is not the case today.*

Reference 4 - 0.86% Coverage

Klokken 16.15 er der fælles briefing.

*At 16.15 there is a joint staff briefing.*

Reference 5 - 1.61% Coverage

Lys m. automatisk dagsindstilling.
Persienner for vinduerne ind til stuerne.
Vigtigt at kunne se tallene på skærmen ude fra computeren.
To store arme, som kommer ned fra loftet med ledninger hængende og flere skærme fastgjort på ”robotarmenes krop”.

*Light with automatic day setting.*

*Blinds for the windows into the living rooms.*

*Important to be able to see the numbers on the screen from outside the computer.*

*Two large “arms” coming down from the ceiling with wires hanging and several screens attached to the "body of the robot arms".*

Reference 6 - 0.29% Coverage

Fællesbriefing
8 sygeplejersker til stede.

*joint staff briefing.*

*8 nurses present*

Reference 7 - 0.85% Coverage

Aftensmad aftales at spises klokken 17.45-18.00 ude på gangen.
Sygeplejerskerne koordinerer, hvem de skal trække på, hvis de får brug for en ekstra mand.

*Dinner is agreed to be eaten at 17.45-18.00 in the hallway.*

*The nurses coordinate who to contact if they need an extra staff.*

Reference 9 - 0.10% Coverage

Medicinrummet

*The medicine room*

Internals\\Empirisk data\\28 feb. Dysfagi + ny patient - § 2 references coded [ 8.75% Coverage]

Reference 1 - 4.89% Coverage

En nye patient ankommer til NISA. Ind ad døren kommer et hold ambulancekører kørerende med en båre, hvor der ligger en ældre kvinde. Sammen med to transportkørere er også to læger med fra afsender-hospitalet, hvor patienten har været ind til nu. De får hende hurtigt flyttet over i hospitalssengen ved at tælle og løfte hende via lagnet. Sygeplejersken fortæller, at hun er ankommet til Silkeborg, hvor hun skal have genoptræning.

*A new patient arrives at NISU. A team of ambulance staff arrives driving a stretcher where an elderly woman is lying. Together with two transport drivers, there are also two doctors from the other hospital, where the patient has been until now. They quickly get her moved over to the hospital bed by counting and lifting her via the sheet. The nurse tells the patient, that she has arrived at Silkeborg Regional Hospital, where she will get rehabilitation.*

Reference 2 - 3.86% Coverage

Efter patientens ankomst og indlogering spørger jeg ude på gangen ind til, hvorfor den ældre kvinde er en NISA-patient. Lægen forklarer mig, at det hele kommer ned til hendes sunkefunktion og det er den, de her på NISA skal sikre, er stabil, før de kan sende hende videre til Hammel. (Minus ordentlig sunkefunktion - slim i lungerne - dysfagi - i værste fald kan det blive fatalt)

*After the patient's arrival and hospitalization, I ask the doctor why the woman is in fact a NISA-patient. The doctor explains to me out in the hallway that it all comes down to her swallow function, and that it is this part they at NISU need to ensure is stable before they can send her on in the system to Hammel (Minus proper swallowing function - mucus in the lungs - dysphagia - in the worst case it can be fatal)*

Internals\\Empirisk data\\30. jan. En snak med udviklingssygeplejersken mandag morgen - § 1 reference coded [ 11.57% Coverage]

Reference 1 - 11.57% Coverage

Videokonference klokken 11. Udviklingssygeplejersken fortæller mig, at sygeplejerskerne skiftes til at gå til mødelokalet, så de kan passe deres patienter. De sparer i disse samtaler med specialister fra Hammel Neurocenter – læger, socialrådgivere m.v. Udviklingssygeplejersken forklarer, at de også løbende har telefonisk kontakt med de ugentlige videokonferencer. Begge dele er vigtige fordi en socialrådgiver måske kan se en god idé, som ikke ville være kommet frem, hvis man kun havde kontakt til én faggruppe.

*Video conference at 11 a.m. The developmental nurse tells me that the nurses take turns going to the meeting room so they can care for their patients. They exchange knowledge and observations during these conversations with specialists from Hammel Neurocentre - doctors, social workers, etc. The developmental nurse explains that they also have regular telephone contact besides the weekly video conference. Both are important because a social worker may be able to see a good idea that would not have come up if they only had contact with one professional group.*

Internals\\Empirisk data\\31. jan. Målsætningssamtale m. S. + familie - § 2 references coded [ 4.42% Coverage]

Reference 1 - 0.51% Coverage

Målsætningssamtale

*Goal setting talk*

Reference 2 - 3.91% Coverage

Den ene terapeut kører en computer ind på et rullebord og tager referat af mødet. De fortæller forældrene og Patient S. at de sender dette til Hammel Neurocenter.

*On a trolley, a therapist drives a computer into the meeting room and takes notes during of the meeting. They tell the parents and patient S. that they are sending the summary to Hammel Neurocentre afterwards.*

Internals\\Empirisk data\\7. marts. En dag med lægen - § 1 reference coded [ 2.25% Coverage]

Reference 1 - 2.25% Coverage

De bruger ordene:

”meget mere stukturet her”

”mere tværfagligt”

”de bruger hinanden”

”de kan lige tage fat i hinanden ude på gangen”

”længere tid til hver patient”

”her "gør man" og prøver noget nyt”

”man er ikke så bange for at prøve nye tiltag”

*They use the words:*

*"Much more structured here"*

*"More interdisciplinary"*

*"They use each other"*

*"They can just grab each other for help out in the hallway"*

*"Longer time for each patient"*

*"Here" we do "and try something new"*

*"We are not so afraid to try new initiatives"*

Internals\\Empirisk data\\9 feb. Ankomst til NISA (patient N.) - § 1 reference coded [ 10.95% Coverage]

Reference 1 - 10.95% Coverage

En terapeut fortæller mig, at nogle ophold på NISA bliver mere eksplicit udtalt som ”vurderingsophold” Alle ophold på NISA er for så vidt vurderingsophold, men nogle er 14 dage, hvor de skal se, om der er nogen fremtid at arbejde videre med.

*A therapist tells me that some stays at NISU are more explicitly pronounced as “assessment stays”. All stays at NISU are in in fact assessment stays, but some patients are more explicit fixed on 14 days, where they have to see if there is any future to work on.*

Reference 2 - 0.47% Coverage

netop også her, i de her omgivelser vi netop kan afprøve alle de her forskellige ting ift. blodtrykket. Altså vi kan, vi kan prøve at få dem op mod stående, og så er vi bare, så har vi jo bare personalet lidt tættere på. Øhm, og hvis det ikke går, så er det jo ned igen, men altså mulighederne byder sig også her, til at vi sagtens kan prøve det. Øhm og der er velvilje til det også, altså lige så vel.

*In these particular surroundings, we can try countless different things while we secure the patients’ blood pressure. We can try to get the patients to stand up, in which case we have the staff [medical] a bit closer by, and if it does not work, we just bring the patient back down again. The possibilities are offered here [at NISU] so that we can easily try things out and there is goodwill for us to do so as well.*

Reference 3 - 0.09% Coverage

Overskrider nogle grænser, som vi ikke ville have gjort før i tiden.

*Exceeds some limits that we would not have done before*
