# Supplementary material for: The temporality of uncertainty in decision-making and treatment of severe brain injury
Source: PLoS One. 2020 Oct 1;15(10):e0238506. doi: 10.1371/journal.pone.0238506 (PMC7529300; doi:10.1371/journal.pone.0238506)
Supplement: S2 File — (DOCX) [file pone.0238506.s002.docx]

Internals\\Empirisk data\\10 marts. Film som metode - § 1 reference coded [ 0.77% Coverage]

Reference 1 - 0.77% Coverage

har fået dem (patienterne) til at vågne mere op

*has made them (the patients) wake up more*

Internals\\Empirisk data\\27 feb - Udskrivelse af P_ - § 1 reference coded [ 0.47% Coverage]

Reference 1 - 0.47% Coverage

usund livsstil, dårlig kost. Svært at nå hende.

*unhealthy lifestyle, poor diet. Hard to reach her.*

Internals\\Empirisk data\\30. jan. En snak med udviklingssygeplejersken mandag morgen - § 1 reference coded [ 1.91% Coverage]

Reference 1 - 1.91% Coverage

”Vi kan ikke redde alle, men vi kan prøve at hjælpe dem” (udviklingssygeplejerske).

*"We cannot save them all, but we can try our best to help them" (developmental nurse).*

Internals\\Empirisk data\\Fokusgruppe - § 2 references coded [ 0.38% Coverage]

Reference 1 - 0.14% Coverage

Vi har til opgave at afdække, hvor det er de (patienterne) har nogle ressourcer, og hvor det er vi kan understøtte dem i deres rehabilitering.

*Our task is to find out where they (the patients) have some resources and where we can support them in their rehabilitation*

Reference 2 - 0.24% Coverage

Netop prøve at finde frem til nogen af de ressourcer, som vi kan bygge videre på. Vi er ret hurtige til at sætte et kortsigtet mål for patienten. Det vil vi gerne gøre det på et aktivitetsniveau som kan måles.

*Try to find some of the hidden resources that we can build on. We are quick to set a short-term goal for the patient. We want to do that at an activity level that can be measured.*
