# Supplementary material for: The temporality of uncertainty in decision-making and treatment of severe brain injury
Source: PLoS One. 2020 Oct 1;15(10):e0238506. doi: 10.1371/journal.pone.0238506 (PMC7529300; doi:10.1371/journal.pone.0238506)
Supplement: S3 File — (DOCX) [file pone.0238506.s003.docx]

Internals\\Empirisk data\\1. marst. Logopæder og målsætningsmøde - § 1 reference coded [ 2.99% Coverage]

Reference 1 - 2.99% Coverage

Fysioterapeuten siger, at det vil hjælpe dem i deres genoptræning, hvis de kender til patient Ks rutiner. De pårørende vil gerne udfylde siderne. De snakker også om, at de gerne må tage en jakke med, når de kommer op på besøg næste gang, så de kan tage hende (patienten) med uden for.

*The physiotherapist says that it will help them in their rehabilitation if they know about patient K's routines. The relatives will help to fill in the pages. They staff also ask the relatives if they will bring a jacket with them next time they come for a visit next so they (the staff) can take her (the patient) outside.*

Internals\\Empirisk data\\10 marts. Film som metode - § 3 references coded [ 14.47% Coverage]

Reference 1 - 6.33% Coverage

Terapeuterne snakker sammen og bliver enige om at starte med skifte patientens (N) T-shirt i stolen, inden de filmer. Det er tydeligt, at det er en del af træningen på den måde, de gør det på. De guider ham til selv at tage over handlingerne ved at sætte en hånd igennem ærmet og ligge hans anden hånd klar til det næste ærme. hvilket han dog ikke formår. Terapeuterne bruger hele tiden N.’s egne hænder.

*The therapists talk together and agree to start by changing the patient's (N) t-shirt in the chair before they begin to film. It is clear that this is part of the training by the way they do it. They guide him to take over the actions himself by putting one hand through the sleeve and laying his other hand ready for the next sleeve. However, he does not manage to take over. Both therapists consequently use N.’s own hands*.

Reference 2 - 0.19% Coverage

Dernæst barbering

*Then shaving*

Reference 3 - 7.95% Coverage

Terapeuten tænder maskinen. Den summer i rummet. Hun rører først hans kind med sin hånd. Hun hjælper med at føre maskinen op til hans kind, da han ikke selv følger hendes opfordring. De barberer hans kind. ”Kan du barberer dig, N?” spørger hun. Hun skubber til ham for at vække ham mere op. På et tidspunkt fører N. selv barbermaskinen op mod sin hårgrænse. Ergoterapeuten stopper ham, og kigger hen på sygeplejersken. De udveksler et blik.

*The therapist turns on the machine. It buzzes in the room. She touches first his cheek with her hand. She helps lead the machine up to his cheek as he does not follow her call. They shave his cheek. "Can you shave, N?" she asks. She pushes to him lightly to wake him more up. At one point, N. leads the shaver up to his hairline. The occupational therapist stops him and looks at the nurse. They exchange a glance.*

Internals\\Empirisk data\\10. feb- observation af første træning for N, 1. forløb - § 8 references coded [ 18.17% Coverage]

Reference 1 - 2.93% Coverage

Omkring tavlen: Fysioterapeuten siger, at de gerne vil have N. til fys for at se, hvad han kan. De vil gerne have ham op ad stå. ”Udfordre ham lidt. Og se hvad der sker”, siger terapeuten. De aftaler, at N. om formiddagen skal have træning. ”Ham vil vi gerne til fra morgentiden. Se hvad han er for én”, siger fys. ”Vi må se, hvad han reagerer på”.

*Around the board: The physiotherapist says to the other staff that they would like to see N. to physio today in order to see what he can do. They want to get him to stand up. “To challenge him a little to see what happens” says the therapist. They agree that N. will get fysio-training in the morning. "We want him from the morning. "We have to see what he reacts to."*

Reference 2 - 2.75% Coverage

Hygiejne inden træningen: Den ene sygeplejerske har fundet nogle vådservietter og siger: ”N. du får lige en klud i ansigtet. Jeg vasker dig, N.”. Hun bruger navnet på patienten hele tiden. Tandbørstning: ”De skal have børstet tænder, N. Den her tandbørste er måske lidt mindre end den du kender hjemme fra”.

*Hygiene before fysio-training: A nurse has found some wet wipes and says: “N. you now get a cloth in your face. I'm washing you, N.”. She uses the name of the patient all the time. Toothbrushing: "They must have brushed teeth, N. This toothbrush is perhaps a little smaller than the one you know at home from".*

Reference 3 - 2.54% Coverage

En fysioterapeut og en ergoterapeut kommer til stuen kl. 9.00, efter at de to sygeplejersker har givet N. medicin, vasket ham med vådserviet, børste hans tænder, renset hans tube-indgang, skiftet hans stomipose.

*A physiotherapist and an occupational therapist enter the ward room at 9.00. Before the two nurses have given N. medicine, washed him with the wet wipe, brushed his teeth, cleaned his tube entrance, and changed his ostomy bag.*

Reference 4 - 2.27% Coverage

”Goddag N. Goddag. Goddag hr. Jeg hedder X. Jeg er fysioterapeut. Sikke nogle arbejdshænder du har dig,” siger fysioterapeuten, der er alene i rummet lige nu, med høj stemme (stor kontrast til den visken der har været på stuen inden da. (Han skal vågne?)).

*Good day, N. Good day, Mr. My name is X. I am a physiotherapist”. Some working hands you got yourself”, says the physiotherapist, who is alone in the room right now, in a loud voice (in stark contrast to the whisper that has been in the ward room up till now. (To get him to wake up?))*

Reference 5 - 0.76% Coverage

”Giv mig et klem i hånden, N. Ja tak. En gang til, så jeg er sikker. Gør det igen. Ja tak!”

*“Give my hand a squeeze, N. Yes, thank you. Do it again so I am sure. Good, N!”*

Reference 6 - 1.24% Coverage

Genoptræningen afsluttes efter en times tid. N. sidder på stuen i en kørestol og får hviletid efter træningen. Han sidder i bar overkrop.

*Rehabilitation ends after an hour. N. sits in the room in a wheelchair and rests after training. He is sitting bare-chested.*

Reference 7 - 2.96% Coverage

Klokken 11: Patienten N. bliver flyttet over i seng og får hvile der. De har lagt ham på venstre side. De har lagt dyner rundt om hans krop. Terapeuten forklarer mig, at det er en del af træningen på NISA, at de skal føle, hvor deres krop er (”starter og slutter”). Mange patienter med hjerneskade har mistet deres fornemmelse af kroppen og dens omkreds.

*11 o'clock: Patient N. is moved to the hospital bed and gets more rest there. They have placed him on his left side. They have put duvets around his body. The therapist explains to me that it is part of the training at NISU that the patients shall feel where their body is ("starts and ends"). Many patients with brain damage have lost their sense of the body and its perimeter.*

Reference 8 - 2.72% Coverage

Klokken 12.30: De to sygeplejerske vender N. om på modsatte side. Den ene fortæller ham, hvad der skal ske, inden de begynder at vende ham. Dynerne tages væk. De skubber ham over med et lagen, ryster ham og trykker på ham.

*12.30: The two nurses turn N. on the opposite side. One tells him what to do before they start turning him over. The duvets are removed. They push him over to the bed with a sheet, push him lightly and press on his body.*

Internals\\Empirisk data\\13. feb. Fys træning m. patient N - § 4 references coded [ 14.33% Coverage]

Reference 1 - 7.15% Coverage

Terapeuten har kørt en briks ind på stuen, som hun stiller ved siden af N.´s seng. Hun tager sine sandaler af og sidder sig op på briksen. Hun ryster i N.´s krop efter at have taget dynen af ham. ”Busker på” siger hun, da hun begynder at tage et par sorte joggingbukser på hans ene ben. Hun skubber til ham flere gange under lænden og kigger på hans ansigt. Ryster ham frem og tilbage flere gange, imens hun giver ham bukserne helt på.

*The therapist has driven a plank bed into the ward room, which she places next to N.´s bed. She takes off her own sandals and sits up on the bed. After taking the duvet off N, she shakes N.'s body. "Trousers on" she says, as she starts to take a pair of black sweatpants on one of his legs. She pushes to him several times under his loins while she looks at his face. Pushes him gently back and forth several times while she gives him his pants completely on.*

Reference 2 - 1.76% Coverage

”Nu på vej ud af sengen N. Du skal denne her vej”, og tager N.´s venstre hånd og kører over briksen overflade.

"*Now out of bed N. You have to go this way", she says, and takes N.'s left hand and runs it over the plank bed surface.*

Reference 3 - 3.65% Coverage

Hun giver N. håndklæde i den modsatte hånd efter vasken. Hun tørrer ham i ansigtet med hånden. Hun observerer en ny reaktion, og kigger igen på praktikanten: ”Så du det blik?”, spørger hun praktikanten. ”Godt gået, N. Jeg tørrer lige efter”.

*She gives N. a towel in the opposite hand after the wash. She wipes his face with her hand. She observes a new reaction and looks again at the intern: "Did you see that look?", She asks the intern. "Well done, N. I dry you a bit".*

Reference 4 - 1.76% Coverage

”N. tak for denne gang. Så er der hvil”, siger terapeuten, imens hun ligger sin hånd på hans pande og stryger den stille.

*“N. thanks for now. Then there is rest”, says the therapist, while she lays her hand on his forehead and strokes it lightly.*

Internals\\Empirisk data\\14. feb. modtagelse af ny patient (P., 2 forl+©b) - § 1 reference coded [ 1.45% Coverage]

Reference 1 - 1.45% Coverage

De to terapeuter bruger sejlet og liften til at få P. over i en kørestol, som står klar. ”P. du får sejl på. Ud af seng. Op at sidde”, siger den ledende terapeut.

*The two therapists use the sail and the lift to get P. into a wheelchair, which is ready. ”P. you get sails on. Out of bed. Up to sit”, says the leading therapist.*

Reference 2 - 0.40% Coverage

træner de området inde i halsen, som ligger ovenover cuffen.

*they train the area inside the throat, which is located above the cuff.*

Internals\\Empirisk data\\18. jan 2017 - anden dag i felten - § 4 references coded [ 12.77% Coverage]

Reference 1 - 3.79% Coverage

Ikke længe efter jeg er kommet, ankommer ergoterapeuten for at træne med patient L.. Hun bruger patient L. egen hånd til at føle på hendes ansigt med. Hun taler til L. imens, og fortæller hende, hvem hun er og hvor L. er henne. Der er ingen reaktion fra L., ud over at hun åbne øjnene og laver grimasser, når det gør ondt, fx under flytningen.

*Not long after I have arrived, the occupational therapist arrives to train with patient L. She uses patient L.'s own hand to feel her face. She talks to L. meanwhile and tells her, who she is and where L. is. There is no reaction from L., except that she opens her eyes and makes grimaces when it hurts, for example during the move.*

Reference 2 - 4.14% Coverage

Ergoterapeuten beder sygeplejersken om at hjælpe med at få flyttet Patient L. fra sengen. De samarbejder om at få Patient L. trukket over på en hård ”seng”, som ergoterapeuten har kørt ind på stuen. Patient L. er overvægtig og ikke let at flytte rundt med. Patient L. har fået støttestrømper – lægen viste mig i går hvordan hun havde vand i benene.

*The occupational therapist asks the nurse to help move Patient L. out of the bed. They work together to get Patient L. pulled over on a hard "bed", which the occupational therapist has driven into the ward room. Patient L. is overweight and not easy to move around with. Patient L. has received support stockings - the doctor showed me yesterday how she had water in her legs.*

Reference 3 - 1.94% Coverage

Efter ergo-træningen er færdig får Patient L. ro til at hvile sig. Sygeplejersken skurer ned for lyset på stuen på det digitale system, og går ud og begynder at udfylde nogle papirer.

*After the ergo training is finished, Patient L. gets peace to rest. The nurse turns down the light in the living room on the digital system, leaves the room and starts filling out some papers.*

Reference 4 - 2.90% Coverage

Senere på formiddagen kommer fysioterapeuten ind til Patient L. Hun taler løbende til Patient L., når hun gør noget. Hun trykker rundt på hendes krop, imens hun hele tiden ser på Patient L.’ ansigt for reaktioner. Når Patient L. oplever det gør ondt, skærer hun ansigter.

*Later in the morning, the physiotherapist comes in to Patient L. She talks to Patient L. ongoing when she does something. She presses her hands around on the patient’s body while constantly looking at L.’s face for reactions. When Patient L. experiences it hurts, she makes faces.*

Internals\\Empirisk data\\2. marts. Vippeleje med K - § 1 reference coded [ 21.66% Coverage]

Reference 1 - 21.66% Coverage

Inde på stuen hos Patient K. bliver der kørt et vippelejet ind af terapeuterne, da det er tid til træning. De skal have K. op at stå. Sammen med sygeplejerskerne får de K. gledet over på briksen fra sin seng, spændt fast med velcroselerne, og i små etaper kører de K. op at stå. Terapeuten forklarer hende, hvad der skal ske, inden hun gør noget. De har hele tiden fokus på hendes blodtryk, men det forbliver fint. K. holder terapeuten i hånden, men virker ikke til at reagere en hel masse på oplevelsen. Da de kører hende ned igen, ligger de hende på en anden briks – pakker hende ind i dyner og puder omkring kroppen, og ligger hende op ad væggen til et hvil.

*In the ward room at Patient K., a special tilt bed is driven in by the therapists, as it is time for training. They will get K. up to stand up straight. Together with the nurses, they get K. slipped onto the plank bed from his bed, fastened her body with Velcro-straps, and in small stages they drive K. up to stand. The therapist explains to her what is going to happen before she does anything. They constantly focus on her blood pressure, but it remains steady. K. holds the therapist in her hand, but it does not seem to react a whole lot to the experience. As they drive her down again, they lay her on another bed - wrap her in duvets and pillows around her body and lay her up against the wall for a rest.*

Internals\\Empirisk data\\21. feb. Røntgen og FEES undersøgelse - § 1 reference coded [ 2.79% Coverage]

Reference 1 - 2.79% Coverage

Fysioterapeuten åbner for den stationære computer og kigger i ”aktivitetsloggen”. Ergoterapeuten har netop skrevet informationerne, som hustruen kom med i går til målsætningsmødet omkring P.´s vaner, glæder og daglige gøremål før hjerneskaden. Fys. siger: ”Det vil give god mening for P. at lave et mål med hunden”.

*The physiotherapist opens the desktop computer and looks in the "activity log". The occupational therapist has just written the information that the wife brought yesterday to the goal meeting about P.´s habits, joys and daily chores before the brain injury. Phys. says: "It will make good sense for P. to make a goal about the dog".*

Internals\\Empirisk data\\24 feb. En dag hvor jeg lærer om hjernen - § 1 reference coded [ 6.38% Coverage]

Reference 1 - 6.38% Coverage

Fysioterapeut tager patient i bad sammen med praktikant som hjælper: Fysioterapeuten forklarer, hvordan det er vigtigt at praktikanten giver N. vaskekluden i hånden for at mærke den. Forsøger at få ham til at lave vaske-bevægelserne som han plejede. Så lægger hun kulden tilbage i hans hænder midt på maven, hvorefter hun rusker ham ved lænden. Dette for at markerer, at den del nu er færdig. ”Så han ikke ligger og tænker: hvor dælen blev kluden af?”.

*The physiotherapist takes the patient in the bath together with the trainee as a helper: The physiotherapist explains to the trainee that is important to give patient N. the washcloth in his hand to feel it. She tries to get him to do the washing moves, he used to know, by himself. Then she puts the cloth back in his hands and then places it on the middle of his stomach, after which she shakes him by the loins. This is to indicate that the part is now complete, she explains. "So that he does not think: where the heck did the cloth go?".*

Internals\\Empirisk data\\24. jan. Fysioterapeut - § 4 references coded [ 21.76% Coverage]

Reference 1 - 9.28% Coverage

Terapeuten tænder for bruseren og får patienten til at mærke strålen af vand og spørger, om det er okay for hende med den temperatur. Patienten synes at give terapeuten et bekræftende svar (ja), men terapeuten siger, at hun godt kan regulere varmen, hvis patienten foretrækker det. Der kommer ikke noget svar tilbage. Terapeuten begynder at vaske håret, efter at have fjernet hår-tørklædet, som sygeplejersken har lagt over ved siden af ved patientens rene tøj. Patienten ligger delvist tildækket med håndklæder.

Hele tiden snakker terapeuten til patienten og forklarer, hvad hun gør. Hun tager patientens hånd og kommer shampoo i den og fører den dernæst på til hendes hår. Patienten begynder at kører hånden rundt oppe i håret, men stopper hurtigt igen. (Hun kan altså her huske, hvad hun skal, når hun vasker hår, men kun når hun bliver ført/ledte førstes skidt på vejen – og ikke særlig lang tid). Samme tilgang bruger terapeuten i forhold til kropsæben, vaskekluden i hovedet og deodoranten og kropscremen efter badet.

*The therapist turns on the shower and makes the patient feel the water and asks, if it is okay for her with that temperature? The patient seems to give the therapist an affirmative answer (yes), but the therapist says that she can regulate the heat, if the patient prefers it. No answer is given. The therapist begins to wash the patient’s hair, after she has removed the scarf, which the nurse has placed next to the patient's clean clothes. The patient is partially covered with towels. The therapist constantly talks to the patient and explains what she is doing. She takes the patient's hand and puts shampoo in it and then applies it to her hair. The patient begins to run his hand around in the hair, but quickly stops again. (She can remember what she has to do, when she washes her hair, but only when she is led on the right track - and not for a very long time). The therapist uses the same approach in relation to the body soap, the washcloth in the face and the deodorant and body cream after the bath.*

Reference 2 - 4.65% Coverage

En sygeplejerske har givet en pårørende grønt lys til at være med til træningen. Sidste gang de prøvede at rejse patienten op, dalede patientens blødtryk hastigt. Derfor er patientens søster i rummet, da vi kommer med vippelejet, og bliver i rummet under hele træningen. Hun får ret bestemt at vide, at hun skal sætte sig i hjørnet i vestre side af rummet.

*A nurse has given a relative permission to be present in the training session. The last time they tried to raise the patient (to get her to stand), the patient's blood pressure dropped rapidly. The patient's sister is in the room, when we arrive with the tilt bed, and stays in the room during the entire training. She is told that she has to sit in the corner on the left side of the room.*

Reference 3 - 5.13% Coverage

Patienten er relativ ung men mor til fire børn. Hun mangler en del tænder. Hun bliver spændt fast og kommer op af stå ved at de bruger hjælpemidlet til at rejse hende lodret op. De bruger noget blåt farvestof, som de giver hende i munden med en finger iført en plastik handske – de skal se om hun kan synke. De tager luften ud af ballonen, som er i hendes hals. De beder hende sige ”ahhh”. Nu bliver søsteren bedt om at komme over. Det er tydeligt, at patienten genkender hende og gerne vil sige noget, men hun ender med at hoste. Hun kan ikke tale (endnu).

*The patient is relatively young but mother to four children. She is missing some teeth. She gets strapped to the tilt lift and is raised to stand up vertically by the tilt lift. The therapists use some blue dye, which they put in the mouth of the patient with a finger wearing a plastic glove - they need to see if she can swallow. They take the air out of the balloon in her throat. They ask her to say "ahhh". Now the sister is asked to come over. It is clear, that the patient recognizes her and wants to say something, but she ends up coughing. She cannot speak (yet).*

Reference 4 - 2.70% Coverage

Vi går ud på trappegangen, hvor hun går op ad ned (89 trin i alt), hvilket er en ny rekord for patienten (

indtil da er rekorden 40 trin fortæller hun. Inden sidste del, fortæller hun, at hun er blevet svimmel og gerne vil ned at sidde. Alligevel går hun ned ad de sidste trin, ind på sin stue og sætter sig på sengen.

*We walk out on the staircase. The patient walks up and down (89 steps in total), which is a new record for her. Until then the record was 40 steps, she says. Before the last steps, she tells that she has become dizzy and would like to sit down. Still, she walks down the last steps, into her ward room, and sits down on the bed.*

Internals\\Empirisk data\\25. jan. En dag med erfaren sygepl. - § 3 references coded [ 14.09% Coverage]

Reference 1 - 1.87% Coverage

Patienten har selv ytret ønske om at komme i bad denne dag. Derfor er det skrevet på tavlen som en aktivitet sammen med fys. om formiddagen efter morgenmad.

*The patient himself has expressed a desire to take a bath this day. Therefore, it is written on the board as an activity along with physiotherapist in the morning after breakfast.*

Reference 2 - 5.72% Coverage

Sammen med fysioterapeuten går jeg ind til Patient J. omkring kl. 11. Fysioterapeuten siger, at de i dag skal ud og prøve at gå på trapper første gange – patienten laver store øjne, men tager imod udfordringen. Hun vil gerne vide, hvor langt de skal for at komme til trapperne. De spørger om hun vil sidde i sin kørestol eller gå med rollator. Hun vælger sidstnævnte. Kørestol kommer med som back up. Fysioterapeuten får Patient J. til at løfte først det ene ben og så det næste og putte det op på trinnet.

*Together with the physiotherapist I walk over to Patient J. around 11 am. The physiotherapist says that today they are going to try to walk on stairs for the first time - the patient makes big eyes, but accepts the challenge. She wants to know, how far they have to get to get to the stairs. The physiotherapist asks if she wants to sit in her wheelchair or walk with a walker. She chooses the latter. Wheelchair comes as back up. The physiotherapist gets Patient J. to lift first one leg and then the next leg and places it up on the step.*

Reference 3 - 6.50% Coverage

Da de har taget første etage, snakker terapeuten med Patient J. om hendes store fremskift og det er tydeligt at Patient J. bliver meget glad (hun smiler stort, hvilket hun sjældent gør). På tre dage har hun udviklet sig fra at kunne gå med rollator, til nu at gå op ad trapper. Terapeuten giver Patient J. ”en stor gulerod”: hvis det går godt med at gå op ad næste etage, må hun selv begynde at gå til og fra toilettet. Det er tydeligt en stor motivation og Patient J. er klar med det samme til at komme af sted.

*As they have done the first floor, the therapist talks to Patient J. about her great progress and it is clear that patient J. is getting very happy. She smiles, which she rarely does. In three days, she has developed from only being able to walk with a walker to now going up stairs. The therapist gives Patient J. "a big carrot": if she can go up the next floor, she will be aloud to walk to and from the toilet by herself. It is clearly a great motivation and Patient J. is ready to leave immediately.*

Internals\\Empirisk data\\27 feb - Udskrivelse af P - § 1 reference coded [ 1.70% Coverage]

Reference 1 - 1.70% Coverage

Patienten har nået mål fra rehabiliteringsplanen om at spise selv, så nu skal nyt mål formuleres. ”Vi skal gå efter det mulige. Hvis hun har været en sofakartoffel, skal vi jo ikke regne med hun bliver en maraton-løber”, påpeger neurologen.

*The patient has reached the goal from the rehabilitation plan “to eat herself”, so now a new goal must be formulated. "We have to go for the possible. If she has been a couch potato, we shall not expect her to become a marathon runner ", the neurologist points out.*

Internals\\Empirisk data\\27 marts (aftenvagt) - § 1 reference coded [ 0.31% Coverage]

Reference 1 - 0.31% Coverage

Da luften er taget af ballonen, kan patient E. pludselig snakke. Jeg bliver meget overrasket.

*As the air is taken off the balloon, patient E. can suddenly talk. I'm very surprised.*

Internals\\Empirisk data\\30. jan. En snak med udviklingssygeplejersken mandag morgen - § 1 reference coded [ 2.21% Coverage]

Reference 1 - 2.21% Coverage

Vi bruger de pårørende til at få svar på spørgsmål, som kan hjælpe os i rehabiliteringen.

*We use the relatives to get answers to questions that can help us in the rehabilitation.*

Internals\\Empirisk data\\31. jan. Målsætningssamtale m. S. + familie - § 1 reference coded [ 6.29% Coverage]

Reference 1 - 6.29% Coverage

Terapeuten forklarer, at det vigtigste for dem er at kigge på Patient S. som ét samlet menneske. De ser helheden, hvorfor CT-billederne ikke altid betyder så meget. Derfor snakker de også mere om det, hun kan gøre og ikke kan, i stedet for at gå ind i billederne fra røntgen.

*The therapist explains that the most important thing for them is to look at Patient S. as a whole human being. They see the big picture, which is why images do not always mean that much. Therefore, they talk more about what she can and cannot do, instead of going into the pictures from the x-ray.*

Internals\\Empirisk data\\Fokusgruppe - § 3 references coded [ 0.95% Coverage]

Reference 1 - 0.14% Coverage

Vores patienter bliver nogle gange presset ud i noget træningsmæssigt, som vi jo ikke havde gjort før i tiden.

*Our patients are sometimes pushed into something training-wise, which we had not done before.*

Reference 3 - 0.72% Coverage

Læge: Jeg stod og så på patienten samtidig med, I gjorde de der ting, og det var pludselig en helt anden patient, fordi det gik jo fra, at det armene bare gik rundt og han slog ud og var motorisk urolig og sådan noget, til at man kunne se, at, at det på en eller anden måde gav en mening for han det i foretog jer. Og der var det, i hvert fald i min hjerne, overbevist om at her var en patient, som i kunne gøre noget for, ik? Og ikke bare var en som lå der og ville blive en grøntsag i virkeligheden, ik? Og det tror jeg det er meget vigtigt. Det viser også meget godt, at det skal være tværfagligt det vi fortager os, ik?

*Doctor: I was standing and looking at the patient at the same time, you (the therapists) were doing those things you do, and it was suddenly a completely different patient. Because it turned out that from his arms just went around and he knocked out and was motor restless and stuff like that to that one could see that it somehow made sense to him, what you did. And at least in my brain it was at this point I was convinced that here was a patient that you could do something for, right? And not just someone who was lying there and wanted to become a “vegetable”. And I think that is very important. It also shows very well that it must be interdisciplinary what we do, right?*
