# Supplementary material for: The temporality of uncertainty in decision-making and treatment of severe brain injury
Source: PLoS One. 2020 Oct 1;15(10):e0238506. doi: 10.1371/journal.pone.0238506 (PMC7529300; doi:10.1371/journal.pone.0238506)
Supplement: S4 File — (DOCX) [file pone.0238506.s004.docx]

Internals\\Empirisk data\\10 marts. Film som metode - § 1 reference coded [ 13.00% Coverage]

Reference 1 - 13.00% Coverage

Sygeplejersken siger, at hun synes det er vildt at høre, at Patient B ligge pludselig bare skulle til Hammel. Hun havde været hos ham fra starten, da han lå med armene i spænd. Jeg siger, at jeg umiddelbart havde troet, at Patient N. ville være kommet sig hurtigere end Patient B. ved bare at kigge på dem. Det kan Sygeplejersken godt forstå. Hun siger, at neurologen dog har sagt fra start, at Patient B. har bedre chancer for at blive bedre end Patient N. ”Neurologen kan bare se noget, som vi andre ikke kan. Det er umuligt at vurdere…” Sygeplejersken siger, at det jo ikke er til at vide, om det er noget de har gjort, der har fået patienten til at vågne mere op eller om det er medicinen eller noget helt andet, ”men det er fedt, når det sker. Men desværre får mange infektioner, som rykker dem tilbage”, fortæller sygeplejersken.

*The nurse tells me that she thinks it's crazy to hear that Patient B is suddenly going to Hammel Neurocentre. She had been with him from the beginning when he lay with his arms all tighten. I say that I thought that Patient N. would have recovered faster than Patient B. just by looking at them. The nurse understands that point. She says that the neurologist, however, has said from the start that Patient B. had better chances of getting better than Patient N. “The neurologist can just see something that the rest of us cannot. It is impossible to assess…" The nurse says that it is not to know if it is something, they have done that has made the patient wake up more or if it is the medicine or something completely different, "but it is awesome when it happens. But unfortunately, many patients get infections that pull them back,” says the nurse.*

Internals\\Empirisk data\\10. feb- observation af første træning for N, 1. forløb - § 9 references coded [ 18.30% Coverage]

Reference 1 - 2.93% Coverage

Omkring tavlen: Fysioterapeuten siger, at de gerne vil have N. til fys for at se, hvad han kan. De vil gerne have ham op ad stå. ”Udfordre ham lidt. Og se hvad der sker”, siger terapeuten. De aftaler, at N. om formiddagen skal have træning. ”Ham vil vi gerne til fra morgentiden. Se hvad han er for én”, siger fys. ”Vi må se, hvad han reagerer på”.

*Around the board: The physiotherapist says to the other staff that they would like to see N. to physio today in order to see what he can do. They want to get him to stand up. “To challenge him a little to see what happens” says the therapist. They agree that N. will get fysio-training in the morning. "We want him from the morning. "We have to see what he reacts to."*

Reference 2 - 1.22% Coverage

Den erfarne sygeplejerske giver Patient N. medicin via hans slager. ”Det er vigtigt, han får noget smertestillende inden træning”

*The experienced nurse gives Patient N. medication via his tube. "It is important that he gets some painkillers before training"*

Reference 3 - 0.76% Coverage

”Giv mig et klem i hånden, N. Ja tak. En gang til, så jeg er sikker. Gør det igen. Ja tak!”

*“Give my hand a squeeze, N. Yes, thank you. Do it again so I am sure. Good, N!”*

Reference 4 - 0.75% Coverage

De ser begge på hans ansigt, imens de løfter hans ene arm op. De bevæger hans ben ud og ind.

*They both look at his face as they lift one of his arms up. They move his legs in and out.*

Reference 5 - 3.25% Coverage

Jeg spørger ind til, hvordan de vurderer, om det er bedst for patienten med hvile eller træning. Terapeuten fortæller, at det kan svært at vurdere om hvile eller udfordringer er bedst for patienten. Det gør det vanskeligt, når de ikke kender patienten så godt. De kigger på mimikken og på patientens værdier på skærmene og ser, hvordan han reagerer på det, de gør.

*I ask how they assess whether it is best for the patient with rest or exercise. The therapist says that it can be difficult to assess whether rest or challenges are best for the patient. It makes it difficult when they do not know the patient so well. They look at the facial expressions and the patient's values ​​on the screens to see how he reacts to what they do.*

Reference 6 - 1.88% Coverage

”jeg tror ikke han har ondt. Jeg tror, han mærker det”. ”Det ligner på ham, at det er lidt irriterende, det du gør”. ”Ja, lidt uvant”, siger de to terapeuter under tandbørstningen (de tror og vurderer hele tiden).

*"I do not think he is in pain. I think he notices it . "It looks at him it's a little annoying what you're doing." "Yes, a little unfamiliar," say the two therapists during the toothbrushing (they “think” and evaluate all the time).*

Reference 7 - 2.32% Coverage

Jeg spørger, om N. ikke kommer til at fryse uden tøj. Sygeplejersken svarer: ”Det er hele tiden en vurdering af, hvad der er bedst lige nu”. Eks. skal hun forstyrre ham i hans hvil for at give en trøje på eller ej. Hun vurderer, at det er bedst lige nu at lade ham sidde og slappe af i bar overkrop.

*I ask if N. is not going to freeze without clothes on his chest. The nurse replies: "It is always an assessment of what is best right now". Shall I disturb him in his rest to give a sweater on or not. She believes that it is best right now to let him sit and relax in bare upper body.*

Reference 8 - 3.83% Coverage

Jeg spørger ind til scoren på 3 fra i går. Terapeuten siger, at vi kan lave en ny i dag. Det gør de tre gange om dagen. Hun krydser af, hvor han nu scorer 10 på det han vidste under træningen. Sidste gang det blev gjort lå den på 5. Terapeuten siger, at det især er, hvis scoren har ligget stabilt på 10 og den så pludselig falder voldsomt, at de skal reagere, men udsving er helt normale. Kommer også an på, hvornår han er mest vågen eller frisk i løbet af en dag.

*I ask to the score of 3 from yesterday. The therapist says we they will make a new one today. They do it three times a day. She crosses off where he now scores 10 on what he knew during training. The last time it was done the score was 5. The therapist says that it is especially if the score has been stable at 10 and it then suddenly drops a lot that they must react, but fluctuations are completely normal. The score also depends on when he is most awake or fresh during a day.*

Reference 9 - 1.35% Coverage

Jeg spørger, hvordan man kan se, om han sover. Den erfarne sygeplejerske siger: ”Vi kender ham ikke så godt endnu”, men at de vurderer det ud fra hans tal.

*I ask how to see if he is asleep. The experienced nurse says: "We do not know him very well yet", but that they assess it based on his numbers.*

Internals\\Empirisk data\\13. feb. Fys træning m. patient N - § 2 references coded [ 17.00% Coverage]

Reference 1 - 8.59% Coverage

Hun har været ved en anden NISA-patient i sidste uge, men der er mange ting, som skal tages hensyn til, når patienterne skal uddeles. Sygeplejersken siger, det giver god mening, at man er ved samme patient over flere dage, længere tid, hvis det kan lade sige gøre. ”Det er jo tit nogle fornemmelser man har [om der sker fremskridt]. Det kan være svært at beskrive sig ud af i en journal.” Hun siger, at ”Klem i Hånd” jo er let at måle, men at det også er en fornemmelse man kan have, om der sker noget, hvis man er ved en patient længere tid.

*She has been with another NISA patient last week, but there are many things that need to be considered when dispensing patients. The nurse says it makes good sense to be with the same patient for several days, longer, if that can be done. "It is often some sort of feeling you have [if there is progress]. It can be difficult to describe to others in a journal.” She says that "Squeeze my Hand" is easy to measure, but that it is also a feeling you can have if something happens if you are with a patient for a long time.*

Reference 2 - 8.41% Coverage

Lægen kommer ind og ser, hvordan det går. Terapeuten fortæller ham, hvad hun har observeret: ”N. åbner øjnene på store stimulationer. I dag også på lyd. Men ingen blikkontakt. …”. Lægen siger ikke så meget, men nikker og anerkender med en ja-lyd. Sammen prøver de ”Klem min Hånd”. Der er bevægelser nede ved hånden. ”Der er mere øjenåbning i dag, end der har været over weekenden”, siger fysioterapeuten. ”De lukker igen ret hurtigt efter stimuli”. Lægen går ud af rummet igen efter 4 minutters tid.

*The doctor enters the room to see how it goes. The therapist tells him, what she has observed: “N. opens his eyes to great stimuli. Today also on sound. But no eye contact. … ”. The doctor does not say much but nods and acknowledges with a yes-sound. Together they try "Squeeze my Hand". There are movements at the hand of the patient. "There is more eye opening today than there has been over the weekend," says the physiotherapist. "They close again pretty quickly after stimuli." The doctor leaves the room again after 4 minutes.*

Internals\\Empirisk data\\13. marts. Kollegas oplæg - § 1 reference coded [ 22.10% Coverage]

Reference 1 - 22.10% Coverage

Vi ved aldrig, hvor lang tid der går…” siger den ene af de to terapeuter. Den anden: ”Det kan være svært at sige lige nu, men når han får de kranieplader på, kan det være det går hurtigt”. Sygeplejersken byder ind og fortæller, at neurologen til videokonferencen netop sagde, at de snart skulle have kontakt til X Hospital for at finde et tidspunkt for N.’s kan få sine kranieplader på igen. De beslutter sig for, at det er nok med ét mål lige nu. Så må de se om tre uger, når den næste rehabiliteringsplan skal udarbejdes. (Der sker her en forhandling i forhold til målet, hvor de byder ind og finder frem til den bedste formulering og mission).

*We never know how long it will take…,” says one of the two therapists. The other: "It is hard to say right now, but when he gets those skull plates on, it may go fast." The nurse tells that the neurologist at the video conference had just said that they soon will contact X Hospital to find a time for N.’s to get his skull plates back on. They decide that one rehabilitation goal is enough right now. Then they will assess in three weeks when the next rehabilitation plan is to be made. (There is a negotiation here in relation to the goal, where they contribute and find the best wording and mission together).*

Internals\\Empirisk data\\14. feb. modtagelse af ny patient (P., 2 forløb) - § 3 references coded [ 4.27% Coverage]

Reference 1 - 1.26% Coverage

P vil score 11 (som er det højeste). ”Når der er så mange [professionelle] ind over, er det den eneste måde, vi kan se, om der sker ændringer”, forklarer hun.

*P will score 11 (which is the highest). "When there are so many [professionals] involved, it's the only way we can see if changes happen," she explains.*

Reference 2 - 0.74% Coverage

”Det er det en score kan – vise udvikling til det bedre eller det værre” (udviklingssygeplejersken).

*"This is what a score can do - show development for the better or the worse" (the development nurse).*

Reference 3 - 2.27% Coverage

en anden terapeut henvender sig til P. og spørger: ”hvordan viser du nej, P.”. Patienten ryster på hovedet. Hun gentager det endnu en gang for at være sikker. ”P. Hvordan viser du ja”. Patienten nikker med små bevægelser. Også dette gøres igen.

*another therapist turns to P. and asks: "how do you show no, P.". The patient shakes his head. She repeats it one more time to be sure. ”P. how do you show yes ”. The patient nods with small movements. This is done again too.*

Internals\\Empirisk data\\15. feb. Tubeskift og stuegang - § 1 reference coded [ 3.37% Coverage]

Reference 1 - 3.37% Coverage

Jeg spørger sygeplejersken, om patienten har fået det bedre i løbet af den uge, han har været her. Det vurderer hun, han ikke umiddelbart har.

*I ask the nurse if the patient has gotten better during the week he has been here. She assesses that he does not on the face of it.*

Internals\\Empirisk data\\16 feb. Målsætningssamtale pårørende til N. + Indscore - § 6 references coded [ 13.41% Coverage]

Reference 1 - 2.08% Coverage

Sygeplejersken fortæller, at der tvivles på afdelingen om, hvorvidt patienten ikke er for dårlig til at blive indstillet videre som NISA-patient. Lige nu er han på et ”vurderingsophold”, for at se om han skal indstille til NISA, men efter hjertestoppet i morges er sygeplejersken i tvivl om, at det kommer til at ske. ”Han er simpelthen måske for dårlig”.

*The nurse says there is doubt in the ward as to whether the patient is not too ill to be referred further as a NISA-patient. Right now, he is on an "assessment stay" to see if he should apply to NISA, but after the cardiac arrest this morning, the nurse is in doubt that it will happen. "He is perhaps simply too bad."*

Reference 2 - 1.76% Coverage

ind-score: handler om at terapeuterne og sygeplejersken gennemgår en masse forskellige scoringstabeller, hvor de på computeren i en tabel har patienten – disse scoringer kan de bruge til at vurdere senere hen i forløbet om patienten har flyttet sig, når de laver næste scoring.

*in-score: is about the therapists and the nurse are going through a lot of different scoring tables, where they have the patient journal on the computer – they can use these scores they to assess later in the process whether the patient “has move” when they make the next score.*

Reference 3 - 2.00% Coverage

De starter fra en ende i mappen og begynder at give P. score fra 1 til 5 på alverdens ting (fx Forståelse, mundstimulering, mimik, hovedkontrol, truksus kontrol, forflytning, ´at stå´, viljebestemt motorik mv.). De snakker om, at der er forskel i scoring på, hvad man gør og hvad man kan gøre.

*They start from one end in the folder and start giving P. scores from 1 to 5 on all sorts of things (e.g. Comprehension, mouth stimulation, facial expressions, head control, trachus control, transfer, ´to stand´, willful motor skills, etc.). They talk about that there is a difference in scoring on what one does and what one can do.*

Reference 4 - 2.09% Coverage

De snakker hele tiden sammen på tværs omkring, hvor de synes P. skal placeres, efter hvad de har observeret inde på stuen. ”Har vi indtryk af, at han forstår det?”, spørger den ene terapeut de andre. I fællesskab bliver de enige om, hvor patienten passer bedst i scoringssystemet. ”Vi er nødt til at tage udgangspunkt i det vi ser lige nu”, siger ergoterapeuten.

*They talk repeatedly together where they think P. should be placed, according to what they have observed inside the ward room. "Do we have the impression that he understands?" One therapist asks the other. Together, they agree on where the patient fits best in the scoring system. "We have to base it on what we see right now," says the occupational therapist.*

Reference 5 - 3.97% Coverage

Da det er første dag sygeplejersken er hos P. er det kun det, som hun har observeret i løbet af formiddagen, og hvad hun kan huske, er blevet fortalt, at hun kan vurdere ham på. Nogle gange, når de er i tvivl, åbner de patientens online journal eller kigger i patientens medsendte papirer for at se, om de han finde noget, som kan hjælpe dem med at placere ham rigtigt. Nogle gange kigger de også ind igennem vinduet ind på N. for at se, hvordan han sidder osv. for at vurdere. ”Hovedkontrol. Det har han.” Han scorer en 3’er i dette punkt. Ved ”at stå”, siger fysioterapeuten: ”Vi har ikke prøvet det, så vi er nødt til at give 1. Vi tester det i morgen”.

*Since this is the first day the nurse is with P., it is only what she has observed during the morning, and what she can remember, has been told that she can assess him on. Sometimes, when in doubt, they open the patient's online journal or look in the patient's accompanying papers to see if they find something that can help them place him the right place. Sometimes they also look in through the window into N. to see how he is sitting, etc. to assess. “Head control. He has that”. He scores a 3 at this point. At "to stand", the physiotherapist says: "We have not tried it yet, so we have to give 1. We will test it tomorrow".*

Reference 6 - 1.51% Coverage

De laver denne scoring 1 gang hver måned pr. patient samt 1 gang inden patienten udskrives fra NISA og sendes hjem eller videre i systemet. Fysioterapeuten siger, at computerprogrammet er lavet sådan, at man kan udskrive en graf over patienternes udvikling.

*They do this scoring once a month per. patient as well as one time before the patient is discharged from NISA and sent home or further in the system. The physiotherapist says that the computer program is made so that one can print a graph of the patients' development.*

Internals\\Empirisk data\\18. jan 2017 - anden dag i felten - § 3 references coded [ 7.42% Coverage]

Reference 1 - 3.27% Coverage

”Glasgow Coma Scale” (GCS), som de bruger til at ranke patientens bevidsthedsniveauet, fx reagerer patienten på lyde osv. Hun siger de ikke har et fast antal gange de skal lave den i timen/dagen, men at de bruger den til at se om patienterne flytter sig. (På akutafdelingerne bruger de skalaen mere end her).

*"Glasgow Coma Scale" (GCS), which they use to rank the patient's level of consciousness, e.g. the patient responds to sounds, etc. She says they do not have a fixed number of times they do it per hour / day, but that they use it to see if patients are in progress. (In the emergency departments, they use the scale more than here).*

Reference 2 - 2.45% Coverage

Fysioterapeuten har svært ved at vurdere, om det er fordi Patient L. bruger al sin energi på at trække vejret igennem tuben (siger hvæsende lyde), eller om spændingerne er en reaktion på hjerneskaden, hvor muskler kan blive stive.

*The physiotherapist has a hard time assessing whether it is because Patient L. uses all her energy to breath through the tube (says hissing sounds), or whether the tension is a reaction to the brain damage where muscles can become stiff.*

Reference 3 - 1.70% Coverage

Sammen med en anden læge bliver det besluttet, at hun skal have skiftet tuben i sin hals til en, der er et nummer større. Hun bruger al sin energi på at trække vejret.

*Together with another doctor, it is decided that she must have changed the tube in her throat to one that is a number larger. She uses all her energy to breath.*

Internals\\Empirisk data\\20 feb. Praktikant og pårørendemøde - § 2 references coded [ 3.64% Coverage]

Reference 1 - 1.52% Coverage

”Vi har prøvet alt mellem himmel og jord”. De har bedøvet ham nu, for at have styr på ham. ”Vi river os lidt i håret for, hvad vi kan gøre ved ham. Derfor gjort ham til NISA, så vores terapeuter kan gå ind og blive set af jer”.

*"We have tried everything." They have anesthetized him now, to keep track of him. "We tear our hair out to figure out what we can do about him. That is why he has been become a NISA-patient, so that our therapists can go in and see to him”.*

Reference 2 - 2.12% Coverage

De vender nogle forskellige muligheder, hvor terapeut spørger ind til patienten og lægen svarer. De aftaler, at terapeuterne kommer ind og ser ham de næste par dage, tager kontakt til kollegaerne fra Hammel og tager ham op igen næste mandag. To sygeplejerske rejser sig og forlader rummet. De går videre til næste patient.

*They talk about some different options, where the therapist asks about the patient and the doctor answers. They agree that the therapists will come and see him for the next few days, contact the colleagues from Hammel and get back to the status of the male patient again next Monday. Two nurses get up and leave the room. They move on to talk about the next patient.*

Internals\\Empirisk data\\21. feb. Røntgen og FEES undersøgelse - § 4 references coded [ 14.79% Coverage]

Reference 1 - 6.30% Coverage

Terapeuten starter med at spørge lægen (nok på vegne af mig og praktikanten) om, hvad formålet med FEES-undersøgelsen er? Lægen svarer, at det er at få status og for at få en idé om, hvornår man vil kunne afcuffe, N. Terapeuten stater med at tage en dims til at suge slim op med igennem Suchen Aiden. Den bliver fyldt helt op. Hun finder endnu en og fylder også den op. ”Hold da op!”, siger lægen. Han siger, at en FEES vil være spild af tid, fordi han allerede kan se ved tilstedeværelsen af alt det slim, at N. på ingen måde er klar til at blive afcuffet. ”Vi kører igen”, siger han. Men ergoterapeuten synes alligevel, de skal lave undersøgelsen. Lægen indvilliger: ”Nu er vi her alligevel allerede”.

*The therapist starts by asking the doctor (probably on behalf of me and the trainee) what the purpose of the FEES-study is? The doctor replies that it is to get status and to get an idea of ​​when they will be able to “decuff”, the patient N. The therapist states to take a “thing” to suck mucus up through the “suction aid”. It gets filled up quickly. She finds another “thing” and fills it up too. "Oh dear!", says the doctor. He says that a FEES will be a waste of time because he can already see in the presence of all the mucus that N. is in no way ready to be “de-cuffed”. "We're leave again," he says. But the occupational therapist still thinks they should do the examination. The doctor accepts: "We are already here anyway".*

Reference 2 - 3.62% Coverage

Næste punkt handler om ”Det hver faggruppe skal gøre for at arbejde hen imod målet”, forklarer fysioterapeuten. Idéen med rehabiliteringsplanen er netop, at alle arbejder hen imod samme mål tværfagligt, forklarer de mig. Sygeplejersken kommer ud fra stuen og tager plads bag de to terapeuter. De gennemgår hurtigt det første mål, som de har lavet, hvilket sygeplejersken synes lyder fint.

*The next point is about "What each professional group must do to work towards the goal", explains the physiotherapist. The idea of ​​the rehabilitation plan is precisely that everyone works towards the same goal interdisciplinary, they explain to me. The nurse comes out of the ward room and takes a seat behind the two therapists. They quickly talk over the first goal, they have made, which the nurse thinks sounds fine.*

Reference 3 - 1.85% Coverage

Terapeuterne fortætter mig bagefter, da jeg spørger ind til formålet og brugen af rehabiliteringsplanen, at de ofte laver et mål omkring tuben og et omkring forflytning, ligesom tilfældet med P. i dag

*The therapists tell me afterwards, when I ask about the purpose and use of the rehabilitation plan, that they often make one goal about the tube and one about transfer, just as is the case with P. today*

Reference 4 - 3.02% Coverage

”Er der noget, du vil tilføje?”, spørger terapeuten sygeplejersken. Hun siger, at det terapeuterne siger, er det samme, som hun har tænkt på og observeret. På baggrund af sygeplejerskens tidligere observationer skriver de, at der skal undersøges om P. kan bruge blok eller staveplade ved at laves en ”udredning af kommunikationshjælpemidler”.

*“Is there anything you want to add?” the therapist asks the nurse. She says that what the therapists are saying is the same thing that she has been thinking about and observing. Based on the nurse's previous observations, they write that it must be investigated whether P. is able to use a block or spelling board by making a "study of communication aids".*

Internals\\Empirisk data\\24 feb. En dag hvor jeg lærer om hjernen - § 6 references coded [ 19.20% Coverage]

Reference 1 - 5.43% Coverage

Lægen spørger den nyansatte sygeplejersken om hun ved om der er indsendt en prøve (”dyrkning”) af det udtræk, der blev lavet på patienten i går af væsken i hans lunger. Hun svarer, at det mener hun ikke der er blevet gjort. Lægen kigger i journalen og ærgrer sig over, det ikke blev gjort. De er på jagt efter, hvad hans forsatte høje infektionstal skyldes, og måske kunne det have givet et svar eller i det mindste udelukket den del af søgningen.

*The doctor asks the newly hired nurse if she knows if a sample of the extract that taking on the patient yesterday of the fluid in his lungs has been sent off. She replies that she does not think so. The doctor looks in the medical record and is annoyed that it has not been done. They are on the hunt for the reason why the patient continues to have high infection rates, and perhaps that could have provided an answer or at least ruled out that part of the search.*

Reference 2 - 1.17% Coverage

Der er sket en udvikling på billeder, som bekymrer overlægen – og måske også overrasker – ham.

*There has been a development on the images that worries the doctor - and perhaps also surprises - him.*

Reference 3 - 4.43% Coverage

Han ser på billederne én gang til og siger: ”Det gør mig svært urolig det her. Det ser ud ad helvedes til. Jeg tror, at jeg ringer til en ven”. Han tager den gule telefon frem og ringer til neurologen fra Hammel Neurocenter. Hun kommer herud senere samme dag. De taler sammen noget tid, hvor lægen forklarer neurologen om hans opdagelse og spørger ind til hendes vurdering.

*He looks at the images one more time and says: “This makes me very worried. It looks like hell. I think I'm calling a friend”. He picks up the yellow phone and calls the neurologist from Hammel Neurocenter. She's coming in later today. They talk for some time, where the doctor explains about his discovery to the neurologist and asks about for her assessment.*

Reference 4 - 1.19% Coverage

Jeg spørger ind til, hvorfor man på det andet sygehus har gjort, som man har, hvortil han svarer: ”Det er jo en vurderingssags”.

*I ask why they at the other hospital have done what has been done, to which he replies: "It is a matter of assessment".*

Reference 5 - 1.86% Coverage

Note til mig selv: Det er nemt at være bagklog. Det handler om at de tager beslutninger og vurdering med den viden de nu engang har på det givne tidspunkt!

*Note: It's easy to be wise after the event. It is about that they are making decisions and assessing with the knowledge they have at the given time!*

Reference 6 - 5.11% Coverage

”Jeg havde bare en fornemmelse af, at han var derinde. Det handlede bare om at kredse lidt i overfladen”. Sygeplejersken ser stadig, at Patient N kommer til at ”kravle rundt på væggene”. Hun fortæller, at det er det ”vi ser i Klinikken”, som vi må tage udgangspunkt i, og ikke hvad testene viser. Hvis en test fx viser, at hans blodtryk er normalt, men vi kan se med vores øjne at noget ikke stemmer, så er det jo det vi skal reagere på.

*"I just had a feeling he was in there. It was just a matter of “scratching a little on the surface””. The nurse still believes that Patient N will "crawl around on the walls". She says that it is we see in “the Clinic" that we must take as a starting point, and not what the tests show. If a test, for example, shows that his blood pressure is normal, but we can see with our own eyes that something is not right, then that is what we must react to.*

Internals\\Empirisk data\\24. jan. Fysioterapeut - § 1 reference coded [ 1.03% Coverage]

Reference 1 - 1.03% Coverage

(Interessant: En ”vurderingssag” fra personale til personale, hvordan de oplever fremgangen hos patienten).

*(Interesting: An "assessment case" amongst the staff, how they experience the progress of the patient)*

Internals\\Empirisk data\\25. jan. En dag med erfaren sygepl. - § 2 references coded [ 1.84% Coverage]

Reference 1 - 1.04% Coverage

Det ser sygeplejersken som et rigtigt godt tegn – at patienten har lært at lytte til sig selv og forstå det.

*The nurse sees this as a really good sign - that the patient has learned to listen to herself and understand it.*

Reference 2 - 0.80% Coverage

Hun viser mig patienternes online journaler - Hele tiden vurderinger

*She shows me the patients' online medical records - All the time assessments*

Internals\\Empirisk data\\27 feb - Udskrivelse af P. - § 2 references coded [ 5.42% Coverage]

Reference 1 - 3.55% Coverage

Jeg spørger ind til, hvorfor terapeuterne skal filme N., da det er første gang jeg er støt på denne metode. Den ene forklarer, at video kan hjælpe dem med at se om medicinen har effekt på patienten. Ved at filme patienten inden han får den, når han lige har fået den, når den er på sit højeste punkt, når det tages væk, kan de altså vurdere, om den har positiv effekt for patientens vågenhed. Det er medicinen ”ritalin”, som er et slags ”opvågningsmedicin”.

*I ask why the therapists are going to film N., as this is the first time, I have encountered this method. One explains that video can help them to see if the medication is influencing the patient. By filming the patient before he gets it, when he has just had it, when it is at its highest point, when it is taken away, they can thus assess whether it has a positive effect on the patient's alertness. It is the medicine "ritalin", which is a kind of "awakening medicine".*

Reference 2 - 1.86% Coverage

”Vi bruger video til at vurdere, om han får noget ud af medicinen eller om han ikke får noget ud af det”, forklarer terapeuten mig. Denne ”Hammel-metode” m. videooptagelser er til at opnå vågenhed og ikke give unødig medicin.

*"We use video to assess whether he is getting anything out of the medicine or not," the therapist explains to me. This "Hammel method" with video recordings is to achieve alertness and not give unnecessary medication.*

Internals\\Empirisk data\\28 feb. Dysfagi + ny patient - § 2 references coded [ 18.19% Coverage]

Reference 1 - 10.82% Coverage

Lægen åbner EPJ og spørger sygeplejersken.: ”Hvad er der sker?”. Hun fortæller overskrifterne: Opstart i ritelin, terapeuterne skriver at der er positiv respons herved. ”Ellers står der ikke så meget”. De starter fra en ende af og snakker om medicinen ritalin, som der er lidt forvirring omkring. Lægen synes ikke at kunne finde, hvor stor en dosis eller hvornår det blev påbegyndt i EPJ, hvilke dage han skal i ”wash out” i forhold til metoden med at patienten filmes. Det er ikke særlig godt dokumenteret i EPJ. De finder frem til informationen i nogle andre dokumenter. Wash out består af 2 hele dage, hvor N. ikke får ritalin så terapeuterne på film mere objektivt kan vurdere, om det har effekt. Lægen: ”Det bedste ville nok være, hvis det var udefrakommende, som kiggede filmene igennem” – give klarer billede af effekten, uden egne påvirkninger fra vores erfaring med ham.

*The doctor opens the EPJ (electronic patient journal) and asks the nurse: "What is happening?". She tells the headlines: Start-up in ritelin, the therapists write that there is a positive response to this. "Otherwise it does not say much". They start from the end and talk about the medicine ritalin, which there is a bit of confusion about. The doctor does not seem to be able to find information about how large a dose or when it was started in EPJ, which days he has to "wash out" in relation to the method of filming the patient. It is not very well documented in EPJ. They locate the information in some other documents. Wash out consists of two full days where N. does not receive ritalin so that the therapists on film more objectively can assess whether it has an effect. The doctor: "It would probably be best if it was outsiders who looked through the films" – in order to give a clearer picture of the effect, without any personal influences from our experience with him.*

Reference 2 - 7.38% Coverage

Mest henvendt til den yngre sygeplejerske, fortæller lægen, hvad han tænker deres umiddelbare strategi skal være for patienten: hun skal tilsluttes respirator, så de er helt sikre på at de begynder det rigtige sted. Bare fordi de har valgt at tage hende af respirator det andet sted, skal de prøve det her, så de er sikre på at undgå dysfagi – så kan det godt være, at han om 2 timer. kan se, at det ikke er nødvendigt, men det er ifølge lægen bedre at teste det, end at køre ud af et forkert spor fra start bare fordi nogle andre fra det tidligere hospital har valgt denne fremgang.

*Mostly addressed to the younger nurse, the doctor tells what he thinks their immediate strategy should be for the patient: she should be connected to a ventilator, so they are completely sure they are starting in the right place. Just because they have chosen to take her off the ventilator elsewhere, should they still try this out so that they are sure to avoid dysphagia - then it may well be that he in 2 hours can conclude that it is not necessary, but according to the doctor it is better to test it, than to go down the wrong path from the start just because some others from the former hospital have chosen this progress.*

Internals\\Empirisk data\\30 jan. Videokonference - § 5 references coded [ 11.29% Coverage]

Reference 1 - 3.14% Coverage

Sidst har jeg noteret, at hun ikke var relevant for Hammel, og der nok ville gå noget tid. Så der er sket noget siden da så? Lægen vurderer der er sket en god udvikling og at hun er klar.

*Last time I have written down in my notes, that she was not relevant to Hammel and there would probably go some time. So something has happened since then? The doctor assesses that there has been a good development and that she is ready.*

Reference 2 - 1.58% Coverage

”Det varer noget tid, inden hun kan komme ud til jer.” Respirator + fordi hun er så tung.

*"It will take some time before she can come out to you." Ventilator + because she's so heavy.*

Reference 3 - 1.49% Coverage

Svært at vurdere kontaktniveauet, men i går fornemmelse af, at patienten i den grad var med.

*Difficult to assess the level of contact, but yesterday feeling that the patient was present to a large extent.*

Reference 4 - 4.39% Coverage

”Det er gået fra at være et vurderingsophold, men det kan være at det skal laves om”. Lægen: vurderingsopholdet gik på hans vægt og derfor nok ikke kunne han rehabiliteres. Nød til at forlænge processen. ”Se det an de næste par uger, hvordan han udvikler sig.”

*"It has gone from being an assessment stay, but it may need to be changed." The physician: the assessment stay went on his weight and if he could be rehabilitated. Need to extend the process. "Observe him the next few weeks to see how he develops."*

Internals\\Empirisk data\\31. jan. Målsætningssamtale m. S. + familie - § 2 references coded [ 15.23% Coverage]

Reference 1 - 8.95% Coverage

Fysioterapeuten fortæller om træningen i dag, og at hun har lavet test med Patient S. (fx skulle hun koge nudler som proces træning. Hun fortæller, hvad der er gået godt og hvilke ting, der stadig kan ske en bedring på (fx den logiske rækkefølge). Terapeuterne i Hammel vil lave samme test senere. Så kan de se, om der er sket en positiv udvikling.

*The physiotherapist talks about the training today and that she has done tests with Patient S. (e.g. she cooked noodles as part of the process training). She tells what has gone well and what things can still be improved (e.g. the logical order). The therapists in Hammel will do the same test later, so they can see if there has been a positive development.*

Reference 2 - 6.29% Coverage

Terapeuten forklarer, at det vigtigste for dem er at kigge på Patient S. som ét samlet menneske. De ser helheden, hvorfor billederne ikke altid betyder så meget. Derfor snakker de også mere om det, hun kan gøre og ikke kan, i stedet for at gå ind i billederne fra scanningen.

*The therapist explains that the most important thing for them is to look at Patient S. as one complete human being. They see the whole, which is why the pictures do not always mean so much. Therefore, they also talk more about what she can and cannot do, instead of going into the images from the scan.*

Internals\\Empirisk data\\6 apr. Tilbagetrækning af klarmeldning - § 2 references coded [ 31.62% Coverage]

Reference 1 - 21.55% Coverage

Hun siger, at det desværre ikke går så godt. "Hun er ikke klarmeldt til Hammel?", spørger jeg. "Ikke længere", svarer sygeplejersken. Hun har lige fået beskeden fortalt. Jeg spørger, hvordan sådan noget bliver vurderet. Hun fortæller, at det er neurologen som har vurderet, at der ikke sker nogle fremskridt, så de har trukket klarmeldingen til Hammel tilbage.

*She says that unfortunately it is not going so well. "She's not ready for Hammel?" I ask. "Not anymore," the nurse replies. She just got the message told. I ask how such a thing is assessed. She says that it is the neurologist who has assessed that no progress is being made, so they have withdrawn the referral* *to Hammel.*

Reference 2 - 10.07% Coverage

Sygeplejersken siger, at det jo er en del af det her, og at det jo heller ikke giver mening at sende en patient, som ikke kan få noget ud af det videre, og tage en plads for en anden som kan.

*The nurse says that it is part of this work, and that it also does not make sense to send a patient who cannot get anything out of it, further in the system, and take a place for someone else who can.*

Internals\\Empirisk data\\6. marts. mandagskonference - § 4 references coded [ 15.64% Coverage]

Reference 1 - 5.23% Coverage

Neurologen siger, at K. ikke tager over på nogen måde, vurderes ikke til at have blikkontakt af neurologen, men siger, at hun har pæne bevægelsesmønstre med den ene arm (vestre arm). Hun vurderer, at ”kvaliteten af bevidsthed er i den dårlige ende”

*The neurologist says that K. does not take over in any way. She is not assessed to have eye contact by the neurologist but says that the patient has nice movement patterns with one arm (left arm). She assesses that "the quality of consciousness is in the poor end"*

Reference 2 - 3.04% Coverage

Sygeplejersken byder ind og siger, at hun så træk i K. ene mundvig to gange i dag, men ”det var ikke så jeg tænkte, at hun er hel med.”

*The nurse says that she saw small “moves” in the one corner of the mouth of K twice today, but "it was not so I thought she was there completely."*

Reference 3 - 4.27% Coverage

Neurolog vurderer K. til: ”lave ende i minimal bevidsthed”.

Lægen svarer: ”Tror du det. Så det er ikke bare fordi, hun ikke ved/forstå, hvad der sker omkring hende?”

Neurolog: ”Det er svært at sige”.

*The neurologist assesses M. to: "low end in minimal consciousness".*

*The physician replies: ”Do you think so. So it's not just because she does not know / understand what is happening around her?”*

*Neurologist: "It's hard to say".*

Reference 4 - 3.09% Coverage

En siger: ”Men nu må vi se, om vi ikke kan få hende af den cuffede tube”
Neurologen afslutter med at sige, at hun bestemt tror på, at der bliver fremgang med K.

*One says: "But now we have to see if we can get her off the cuffed tube". The neurologist concludes by saying that she certainly believes that there will be progress with K.*

Internals\\Empirisk data\\7. marts. En dag med lægen - § 1 reference coded [ 3.10% Coverage]

Reference 1 - 3.10% Coverage

Jeg stiller lægen spørgsmålet: men hvad hvis, det var blevet vurderet af en anden læge, og han ikke lige havde set den lille forskel, som gjorde han fik en chance - så ville han aldrig være sendt til Hammel som i går. Det giver lægen mig ret i. Han siger, at der til en vis grad er "flydende retningslinjer" - og at det for ham også er nødvendigt til en vis grad.

*I ask the doctor the question: but what if, it had been assessed by another doctor and he/she had not seen the small difference that made him get a chance - then he would never have been sent to Hammel like yesterday. The doctor agrees with me. He says that to a certain extent there are "fluid guidelines" - and that for him it is also necessary to a certain extent.*

Internals\\Empirisk data\\9 feb. Ankomst til NISA (patient N.) - § 1 reference coded [ 8.34% Coverage]

Reference 1 - 8.34% Coverage

Lægen laver første tjek. Ser ham ind i øjnene med lys, kører forsigtigt fingre over tape på hans for hoved, laver klem i hånd test. Giver ham scoren 3 (den laveste score).

*The doctor performs the first check. She looks him in the eyes with a flash light, gently runs her fingers over the tape on his forehead, does “squeeze my hand” test. She gives him the score 3 (the lowest score).*

Internals\\Empirisk data\\Fokusgruppe - § 29 references coded [ 5.69% Coverage]

Reference 1 - 0.55% Coverage

Så det er at bruge min erfaring inden for neurofeltet sammen med intensiverfaringen. Og i første omgang at kortlægge patientens sygdom: Hvad kan vi forvente af patienten og være med til sådan at udstikke planerne, i hvert fald i den første omgang. Hvor det meget er intensivterapien, der fylder mere end det er neurorehabilitering. Ligesom at afstemme, hvad er planen for den del af det.

*So it's using my experience from the neuroscience along with the intensive experience. And in the first place to map the patient's disease: What can we expect from the patient and help to set the plans, at least in the first place. Sometimes it is intensive care that takes up more space than it is neurorehabilitation. So to tune in what is the plan for that part of it.*

Reference 2 - 0.57% Coverage

… at lave en generel undersøgelse af deres funktionsniveau, afdække hvor det er de har nogle ressourcer, og hvor det er, vi kan understøtte dem i deres rehabilitering. Det gør vi jo i samarbejde med sygeplejersker og ergoterapeuter og læge, hvor vi finder ud af, hvad der er den bedste måde at arbejde videre på - om det er omkring kommunikation, omkring afcuffning, omkring hovedkontrol eller omkring at kunne udføre nogle ADL-aktiviteter.

*… To make a general examination of their level of function, uncover where it is they have some resources and where it is we can support them in their rehabilitation. We do this in collaboration with nurses and occupational therapists and doctors, where we find out what is the best way to work onwards - whether it is about communication, about “de-cuffing”, about head control or about being able to perform some ADL-activities.*

Reference 3 - 0.96% Coverage

Vi jonglerer jo egentlig lidt i mellem det intensiv-speciale og rehabilitering, og begge dele skal vi kunne tilgodese. Der er jo meget forskel på … Hvor intensivpatienten er og hvilke opgaver vi har, og det er noget af det, som vi vil skulle kunne jonglere i [En telefon ringer]. Det er vigtigt for patienten, at vi kan se hvad, der er vigtigt i dag: Er det noget respiratorisk, der overskygger rehabiliteringen, eller noget andet. Der kan være mange hensyn at tage og mange vurderinger i løber at dagen, og vi er der jo hele døgnet, som sygeplejersker. Så derfor har vi jo et godt indblik i, hvad patienten formår, og det skal vi så kunne videre give til resten af personalegruppen, så det kan gavne det tværfaglige samarbejde.

*We are in fact juggling a bit between the intensive specialty and rehabilitation, and we must be able to accommodate both. There is a lot of difference between… Where an intensive-patient is and what tasks we have, and that is something that we will have to be able to juggle between [A phone rings]. It is important for the patient that we can see what is important today: Is it something respiratory that overshadows the rehabilitation, or something else. There can be many considerations to take and many assessments in the course of a day, and we are here around the clock, as nurses. So therefore, we have a good insight into what the patient is capable of, and we must then be able to pass this on to the rest of the staff group, so that it can benefit the interdisciplinary collaboration.*

Reference 5 - 0.67% Coverage

Nogle gange, når vi har de her dårlige patienter, som får meget stærkt medicin eller sederende medicin, så er det netop måske ikke altid, det er inde ved patienten at terapien foregår. Det kan også være udenfor. Altså, det er simpelthen en snak om, hvad der er bedst og mest hensigtsmæssigt for den enkelte patient, og hvad de profiterer af og hvad de ikke gør. Det kan netop være at lave en neuro-pædagogisk strategi ift. tilgangen derinde [ved patienten]. Hvordan vi bedst kan understøtte i at måske få ud trappet fra den her medicin.

*Sometimes, when we have these bad patients who receive very strong medication or sedative medication, then it may not always be at the patient that the therapy to takes place. It can also be outside. So, it is simply a talk about what is best and most fitting for the individual patient and what they benefit from and what they do not. It may just be to make a neuro-pedagogical strategy in relation to the approach [for the patient]. How we can best support them, in perhaps getting them off the medicine.*

Reference 6 - 0.16% Coverage

Vi kan ikke se deres potentiale i rehabiliteringen i samme grad, som hvis at de ikke var påvirket af den slags medicin (3.0).

*We are not able to see their potential in rehabilitation to the same degree as if they were not affected by that kind of medication (3.0).*

Reference 9 - 1.00% Coverage

Jeg vil sige ift. beslutningstagen, der er jeg også meget enig med X i, at den er ikke ret svær. Vi har jo også de her tværfaglige score, hvor vi en gang om måneden skal score ift. nogle meget specifikke ting. Og det kan vi jo lidt sort på hvidt se, har der været en udvikling eller har der ikke. Nogle gange så er den ikke helt [så sort-hvid], fordi det er jo ikke de samme der sidder og score. Selvom det er en objektiv score, så bliver man jo lidt subjektiv i, hvordan man oplever patienten. Men sådan generelt set, så vil man kunne se, om der har været fremgang eller om det har stået stille. Og det at vide, at der altid er muligheden for en genhenvisning, hvis der skulle være noget spontan bedring, synes jeg også giver en noget, at man ikke bare afskriver.

*I would say in relation to the decision-making, I also very much agree with X that it is not very difficult. We have these interdisciplinary scores, where we have to score once a month in relation to some very specific things. And here we can see in black and white, if there has been a progress or not. Sometimes it is not quite [so black and white], because it is not the same people who score. Even though it is an objective score, you become a little subjective in how you experience the patient. But in general, you will be able to see if there has been progress or if it [the patient] has stood still. And knowing that there is always the possibility of a referral, if there should be any spontaneous improvement, I also think gives one something that one does not just write off.*

Reference 10 - 0.27% Coverage

Vi kan kun vurdere ud fra det, vi ser. Og ud fra de undersøgelser vi har lavet, ud fra de undersøgelser fysioterapeuten har lavet, ud over de observationer sygeplejerskerne har gjort. (3.0). Så kan vi jo kun agere, som vi gør.

*We can only assess based on what we see. And based on the examinations we have made, based on the examinations made by the physiotherapists, and from the observations made by the nurses. (3.0). Then we can only act as we do.*

Reference 11 - 0.02% Coverage

Kommunikation

*Communication*

Reference 12 - 0.02% Coverage

reagerer patienten

*Does the patient respond*

Reference 13 - 0.01% Coverage

Mimik

*Mimic*

Reference 14 - 0.04% Coverage

efterkomme små opfordringer

*comply with small requests*

Reference 15 - 0.01% Coverage

Stimulus

*Stimulus*

Reference 16 - 0.01% Coverage

Scoringer

*Scores*

Reference 17 - 0.03% Coverage

Tværfaglige scoringer.

*Interdisciplinary scores.*

Reference 18 - 0.05% Coverage

tegn på kommunikation - kontakt.

*signs of communication - contact.*

Reference 19 - 0.01% Coverage

Værdier

*Values*

Reference 20 - 0.01% Coverage

Målinger

*Measurements*

Reference 22 - 0.01% Coverage

Deltagelse

*Participation*

Reference 23 - 0.01% Coverage

Vågenhed

*Alertness*

Reference 24 - 0.24% Coverage

hvor meget de selv kan deltage i badet, og hvornår man kan lade dem selv tage over, hvis de begynder at vise det mindste tegn til at vide, hvad det er de har, altså at de fx får en vaskeklud i ansigtet.

*how much they can participate in the bath themselves, and when you can let them take over themselves, if they start to show the slightest sign of knowing what it is, they have - i.e. if they get a washcloth on their face, for example*.

Reference 25 - 0.06% Coverage

Vi guider dem og ser om de egentlig kan tage over,

*We guide them and see if they can take over,*

Reference 26 - 0.06% Coverage

Det er hele tiden de der små observationer

*It's all the time those little observations*

Reference 27 - 0.01% Coverage

Kropssprog

*Body language*

Reference 28 - 0.25% Coverage

Tværfaglige konferencer, videokonferencer: der evaluerer vi jo også hele tiden på, hvad har virket og hvad har ikke virket.

*Interdisciplinary conferences, video conferences: here we also constantly evaluate what has worked and what has not worked.*

Reference 29 - 0.13% Coverage

Vi bliver nødt til at følge den enkelte patient: hvad de har brug for, og hvornår på døgnet de har brug for det.

*We need to follow the individual patient: what they need and when in the day they need it.*

Reference 30 - 3.13% Coverage

Der vil jeg gerne lige sige, at vi har jo som hver faggruppe har nogen forskellige mål for patienten. Vi sætter dem efter nogle kriterier, som skal være målbare. Vores er på en aktivitet, som vi kan gentage igen og igen og igen og igen og igen. Så selvom patienten har en dårlig dag, så vil vi kunne prøve aktiviteten næste dag. Jeg har aldrig oplevet, at den [beslutningen] har været forskønnet eller at jeg har følt mig…. jeg har altid haft min dokumentation at kigge tilbage på. Vi har evalueret mange gange på den her ting, som vi har sat som et forholdsvis simpelt mål til en start, og har der været en eller anden form for fremgang, så, så ville man opdage det.

*There I would just like to say that we have, as each professional group, some different goals for the patient. We set them according to some criteria, which must be measurable. Ours is on an activity that we can repeat over and over and over and over and over again. So even if the patient has a bad day, we will be able to try the activity the next day. I have never experienced that the decision has been rushed or felt like... I have always had my documentation to look back on. We have evaluated many times on these matters, made a relatively simple goal from the start, and if there has been some kind of progress, then we would have discovered it.*
