# Supplementary material for: The temporality of uncertainty in decision-making and treatment of severe brain injury
Source: PLoS One. 2020 Oct 1;15(10):e0238506. doi: 10.1371/journal.pone.0238506 (PMC7529300; doi:10.1371/journal.pone.0238506)
Supplement: Attachment — Submitted filename: Response to reviewers.docx [file pone.0238506.s005.docx]

Dear Editor and reviewers,

Thank you for your respond and invitation to the resubmission of a revised manuscript previously entitled: *Time and Negotiation of Futures in the treatment of severe brain injury: An ethnographic study of anticipatory action in early neurorehabilitation*

New title:

*The temporality of uncertainty in decision-making and treatment of severe brain injury. An ethnographic study of anticipatory action in early neurorehabilitation*

We would like to thank the reviewers very much for their positive remarks and constructive suggestions to improve the article. The article has now undergone major revision.

Please find our responses to the comments below.

All changes are marked in yellow in the main document.

Best wishes

*Mia Krogager Mathiasen and Hanne Pallesen*

| Reviewer #1: | respond |
| --- | --- |
| Line 46: therefore not therefor | Corrected line 46 |
| Line 57: I would favor defining the ICU abbreviation in line 51 rather than line 57 | Deleted line 57 and move to line 51 |
| Please be sure that the manuscript adheres to the appropriate EQUATOR network guidelines and provide citation. Likely: O'Brien BC, Harris IB, Beckman TJ, Reed DA, Cook DA. Standards for reporting qualitative research: a synthesis of recommendations. Acad Med. 2014;89(9):1245-1251. | The manuscript is now following EQUATOR Guidelines and correct citations are made |
| Please state if the study was approved by an IRB. If so, state the organization whose IRB provided approval, and list the approval number in parentheses. | The study was approved. Missing data added lines 282 - 285 |
| Please state if informed consent was obtained and from whom. Was surrogate consent allowed? | Added lines 285-288 |
| Please state if consent covered both study participation and publication of de-identified aggregate findings. | Described in lines 285 - 288 |
| Line 182-183: Please update software reference: NVivo qualitative data analysis software (QSR International Pty Ltd, Doncaster, Australia) | Added in lines 221- 222 |
| Line 199: defining NSIU should be done back up on line 58 | Changed |
| Please streamline the methods. Please describe criteria for deciding when no further sampling was necessary (e.g., sampling saturation).  Please describe techniques to enhance trustworthiness and credibility of data analysis (e.g., member checking, audit trail, triangulation); rationale. | The section of methods has been streamlined (lines 123-167) and information of sampling saturation (lines 168-170) and how to enhance trustworthiness and credibility of data analysis has been described in lines 170- 178 |
|  |  |
| Reviewer #2: The article addresses an important an important topic - underlying logics of early neurorehab - using an appropriate and under-utilised approach - ethnography. In general, the paper is clearly written, laid-out well, and provides relevant information to the reader. However, I have a number of queries which may need to be addressed in a re-framing of the paper. These are outlined below: | We do appreciate the general impression and the queries in need of been addressed in a re-framing and focusing paper. |
| - The theoretical contribution of the paper is somewhat blurred. While the abstract states the paper investigates how concepts of time play out and help us understand underlying logics and dynamics of of social processes. There is also mention of 'temporal strategy games'. However the bulk of the paper focuses more broadly on the nature and objectives of early neurorehab rather than an in-depth look at the social dynamics or concepts of time. More about time in the macro sense (past, present, future) than micro temporal strategies of staff. - Rather than concepts of time, I found the focus of the paper more on the slightly different - but also very interesting -uncertainty temporalities/alternative futures of clinical actions and decision-making in early neurorehab | We are in the revised paper focusing on the temporality of uncertainty and decision making  The changes in relation to this focus are now pervasive throughout the article.  See: new title + lines 21-22, 72, 181, 216, 228, 233-238, 295- 303, 608-611, 636, 689-699  We have also added more data in the macro sense of strategies and conflicts of staff. Lines 358 - 383 |
| - Methods: Considering the ethnographic approach, I found the methods section lacking in a number of area: | This section has undergone major revision |
| o How were observations ‘participant’ as opposed just observation? [see Raymond Gold's typology] | Line 120: The word *participant* is deleted and the reference changed to Gold, R. (1958) |
| o How many hours observation? | Information added line 152 |
| o Needs a reflective piece on impact of who did observations? | Information added, see line 124 + 142-150 |
| o Was there any iteration between what you set out to observe and how data collection and analysis proceeded? | Information added 123-127 |
| o How many ethnographic interviews were done and with who? There is a mentioned of transcribed interviews (p9) not sure how evident these are in findings | Information added line 151-158 |
| o Why focus group? How long? Discussion topics? | Section added line 158- 167 |
| o Are relatives in interviews or observations? ‘Participated in three meetings with family members’ (p7) how? | Changed the words participated in to observed in order to clarify, line 138. |
| o Page 9 mentions hospital practice documents – not visible in findings | Deleted |
| o Not always clear where data in findings is from (obs, interview, FG) | Added information, lines 348, 533, 626-627, 634, 671 |
| o Integration of data not clear – focus group dialogue appears in abrupt manner | Clarified and changed |
| o Triangulation of data not evident (line 736) | Now described in lines 168- 178 |
| - Linked to the above point, I have some concern about objectivity of analysis. At times analysis feels like it is selling NISU. See lines 244, 301-312, 400-403, 455-456, 616, 723-725, 749. | Thank you very much for this important point. We have looked into the mentioned lines and addressed the points as objectively as possible giving the situation that NISU is a single unit in the Danish healthcare system |
| - Context section on NISU is far too long. Space of Opp section talks a lot about physical space but this doesn’t really feed into key findings – what does this section tell us, other than description of environment – which is then not taken up in discussion. No real discussion of time here either i.e. how time is entangled in physical space isn’t immediately obvious. Findings and discussion focus more on the decisions making and clinical action. | The context section is significantly shortened |
| - Findings structured by; ‘space of opportunities, the clinical work, the assessment of potential’ which I didn't find very informative. An opportunity was missed here for deeper investigation of very important and theoretically rich topics which are only touched on. These include: decision-making rationalities, uncertain futures, how different disciplines negotiate uncertain futures – use of scoring/tools to navigate and manage uncertainty and ‘making the final call’ (564), responsibilities of final call between anaesthesiologists v other HCPs, embodied memories, active v passive rationalities of care (lines 460-463). All of these could be the main focus of the paper (rather than temporal strategies) - as they provide a richer account of the 'social processes' behind the practical clinical work. | We do find that the initial descriptions of NISU and the clinical practice must emerge in findings partly to allow the inexperienced reader with less knowledge of intensive work to gain a sufficient understanding. Furthermore, in terms of the fact that the NISU unit is singular in the Danish healthcare system, but also unusual in the international context, the descriptions are important.  However, we have added some sub-categories and added Figure 1 to give an overview of Findings and discussion. |
| - Must of the key findings and discussion are from the clinical work section – could focus here for re-framing of paper. | We see your points and agree. We have tried to reframe the paper accordingly |
| - While relatives are ‘crucial pieces’ (757), there is little emphasis on them in the findings. | This sentence has been removed from the conclusion |
| - Discussion doesn’t follow through on potential contributions – how uncertainty is managed via situated/alternative futures. Overall, this paper could be much more concise in honing in on how staff manage the negotiation of futures which underpin early neurorehab clinical work. | Content order in the discussion section has been changed to match order and content in findings and the aim of the study |
| - The link to theories in the literature are very broad, using concepts from mobile tech, history and political economy. There is little to no mention of a range of ethnographic healthcare (including intensive care) literature (see below). Could still use ‘anticipatory action’ concept but this need to be integrated more into medical/healthcare literature. | Thank you for enlightening us about relevant ethnographic healthcare literature. The literature is interesting, and one study has been included in the discussion section: Higginson IJ, Rumble C, Shipman C, Koffman J, Sleeman KE, Morgan M, Hopkins P, Noble J, Bernal W, Leonard S, Dampier O, Prentice1 W, Burman R and Costantini M. The value of uncertainty in critical illness? An ethnographic study of patterns and conflicts in care and decision-making trajectories. BMC Anesthesiology (2016) 16:11DOI 10.1186/s12871-016-0177-2 |
|  |  |
| - Overall, I found this paper to be an interesting read of an important topic. It uses a suitable, and under-used, approach in ethnography. However, I feel the paper needs a major re-frame to cut unnecessary text, add in methodological rigor for ethnographic approach, and frame the paper around the uncertainties of decision-making and how this is managed by staff in different positions. Also maybe more on role of family in this process.  Best of luck! | Thank you for these remarks. We hope that the revised paper now appears more focused and methodically solid |
