# Supplementary material for: The temporality of uncertainty in decision-making and treatment of severe brain injury
Source: PLoS One. 2020 Oct 1;15(10):e0238506. doi: 10.1371/journal.pone.0238506 (PMC7529300; doi:10.1371/journal.pone.0238506)
Supplement: Attachment — Submitted filename: Response letter to reviewers.docx [file pone.0238506.s006.docx]

Response letter to reviewers

Dear Editor and reviewers,

Thank you for your replies. We hereby resubmit a revised manuscript with the title: The temporality of uncertainty in decision making and treatment of severe brain injury. An ethnographic study of anticipatory action in early neurorehabilitation

We would like to thank the reviewers for the positive remarks and constructive suggestions to improve the article further. Please find our responses to the comments below.

All changes are marked in yellow in the main document.

Best wishes

Mia Krogager Mathiasen and Hanne Pallesen

| Reviewer #1: | respond |
| --- | --- |
| Rather than stating “first author”, consider defining Principle Investigator as PI and using that abbreviation thereafter | We have made the recommended change to P1 |
| - The importance of the science gets lost in all the storytelling and extraneous prose. This manuscript needs to be re-structured and significantly shortened to meet journal standards/format and to keep the reader engaged and “on task”. | The ethnographic descriptions have been shortened throughout the manuscript (approx. one page in total). |
| Line 25: please be consistent with the explained definition of NISU that is used in the main text (neuro-intensive step-down unit) | Changed |
| - Line 26-28: Wording is a little confusing. Is the author intending to say: … negotiation of futures takes place in the modern ICU by strategically building upon past experiences. | The sentence has been modified |
| - Line 31: present, not presence | Changed |
| - Line 31: consider rephrasing to … and desired futures. | We have made the recommended change and deleted ‘certain hoped-for futures’ |
| - Introduction: In order to make the writing more concise, consider the following revision (not mandatory). I would advise starting the Introduction as follows (your choice): From the onset of a neurologic insult (e.g. traumatic brain injury, stroke, etc.) the patient’s experience of time and space changes [1, 42 2, 3]. Conversely, a clock begins from the health provider perspective, during which many believe an early window exists in which the brain's dynamic response to injury is heightened and rehabilitation is assumed to be particularly effective. | We have made the recommended change |
| - Consider abbreviating severe acquired brain injury as this statement is used frequently. | We have made the recommended change (sABI) |
| - Line 103: has demonstrated | Changed |
| - Line 143: extent (not extend) | Changed |
| - The methods contain too much extraneous text and discussion for a medical journal. This needs to be tightened up and condensed. The theoretical framework (lines 179-217) should be condensed and included in the end of the introduction (before methods). Much of it could be removed. | The section has been tightened up and condensed (deleted lines: 129-130, 172 175, 175 – 180, 120-122, 122- 130)  We have chosen to keep the location of the theoretical framework. We find that the framework for the following analysis stands more clearly in this way. |
| - Line 142-150: I would recommend condensing this passage as follows: The primary investigator dressed in attire similar to other healthcare workers and wore a badge identifying the researcher role. The observer role proved non-disruptive to the teams, which are accustomed to those in observer roles including students and external providers. | We have made the recommended change |
| - Line 153: conversations, not conservations | Changed |
| - Line 155: would it be more accurate to say surrogate, spouse, or family/friend, rather than wife? Specifying wife implies that all participants were married men (or lesbians). What if the person was un-married, or woman? | Changed to “spouse” |
| - Line 161: Was 1h 36min an average amount of time. It seems like an odd amount of time to specify a priori. | Changed to “about 1.5 hours” |
| - Line 168-170: Sampling saturation occurred after 3 months of fieldwork. | Modified, line 188-189 |
| - Line 170: ...credibility, the processes | Changed |
| - Line 170-172: ...generation were discussed and clarified during regular research team meetings, where PI analyses were validated by co-investigators. | Changed |
| - Line 173: endeavored | Changed |
| - Line 176: there are 2 sets of parentheses, so the sentence should end: ...theories)). | Changed |
| - Line 176-178: sentence fragment, please revise. | The sentence has been revised, line 196 line |
| - Line 228-237: not necessary, would delete. This is still the methods. The findings are presented later. | Deleted |
| - Context, Line 238-279: Should be condensed and moved up early in methods when discussing study environment and patient population. | Condensed. The recommend lines have been deleted and moved up as recommended to line 134 |
| - Line 284-285: The chosen verbage leaves the statement open to interpretation. I would favor just stating: Informed consent by a designated health care surrogate was permitted in cases where the patient did not have decision-making capacity. | Changed |
| - Line 292-294: no need to state what you are “going” to say in the results. Just state the results. I would delete these sentences. | The sentences are deleted |
| - Results: Please start by stating the number of patients screened for study, the number consented, and the number included in the final analysis. Then state reasons for exclusion with patient numbers. | Information has been added in the section “Data collection”, line 181-187. |
| Reviewer #2: |  |
| Revised edition is much improved. Methods much stronger. Appreciate that English may not be first language but there are some small grammatical errors throughout which may need to be picked up by editors. Have flagged as many as I could below: | Thank you for your comment about the revised edition and for your help with the grammatical errors. |
| Line 22: delete ‘is’ | Error is corrected |
| Line 30: ‘presence’ should be present | Error is corrected |
| Line 111: delete particular | Changed |
| Line 112: observations | Error is corrected |
| Line 122: intensive | Error is corrected |
| Line 139: extent | Error is corrected |
| Line 144: ‘stood aside’ to accompanied | Sentence has been changed |
| Line 147-152: re: data collection – for clarity could state outright that analysis is based on 130 hours of observation, ?? formal (i.e. recorded) semi-structured interviews (if any or were they all informal, part of observational fieldwork? If so, state that they were informal, handwritten), and 1 focus group…. | The sentences have been clarified |
| Line 165: ….generation were discussed… | Changed |
| Lines 167-171: is there a reference for use of data, investigator and theoretical triangulation? | Reference added, line 197 |
| Line 206: delete ‘for’ | Deleted |
| Line 211: interviews “and” observation | Error is corrected |
| Line 215: also focus groups? | Added |
| Line 257: remove bracket? | Removed |
| Line 284: is presented | The sentence is been deleted |
| Line 289: should be ‘main’ and ‘analysis’ | Changed, line 264 |
| Line 305: check patient | Changed |
| Line 349 nurses who expressed | Changed, line 432 |
| Line 346-370: really interesting discussion from interviewee about advantages and disadvantages of NISU set-up. Almost seems like it might be better for patients but slightly worse for her in terms of her experience of professional autonomy. Could consider whether this inter-disciplinary impinging on autonomy experience would be better placed in clinical work or interdisciplinary sections? | The discussion has been moved to The interdisciplinary section, line 429 - 454 |
| Line 379: pleasant future life | Changed, line 323 |
| Line 482: Inter-disciplinary Collaboration? | Changed to The clinical interdisciplinary collaboration, line 409 |
| Line 509: delete ‘see next chapter’ | Deleted |
| Line 549: sentence may not be needed, previous sentences provide link to following section | The sentence is been deleted |
| Line 632-652: really interesting data – highlights how scores and navigational tools provide some sort of rationale (perhaps comfort for professionals) but the interdisplinary nature of decision-making means this is still a social process – shaped by other professionals’ opinions – this could be added to discussion (lines 805-808) re: ethical dilemmas of negotiating future lives. | Added in line 755 – 759. |
| Line 676-686: some repetition – intro of three logics not needed in both places. Suggest just at start of discussion. | Deleted |
| Line 740-742: sentence meaning unclear | We have not been able to locate the sentence |
| Line 812-814: not sure what this means – perhaps better to say that some of this practices in ICU and neuro-rehab are common and therefore findings may inform research in other settings? | Deleted |
| I think the literature and discussion would benefit from the addition of one or 2 more socially focused papers – as the findings are about the social dynamics and interprofessional collaboration of neuro-rehab e.g. Andreas Xyrichis, Karen Lowton, Anne Marie Rafferty (2017) Accomplishing professional jurisdiction in intensive care: An ethnographic study of three units - https://doi.org/10.1016/j.socscimed.2017.03.047 | Addition added line 760-768 |
